# Identification of female-enriched and disease-associated microglia (FDAMic) contributes to sexual dimorphism in late-onset Alzheimer’s disease

**DOI:** 10.1186/s12974-023-02987-4

**Published:** 2024-01-04

**Authors:** Deng Wu, Xiaoman Bi, Kim Hei-Man Chow

**Affiliations:** 1 School of Life Sciences, Faculty of Science, The Chinese University of Hong Kong, Hong Kong SAR, 999077 China; 2 Gerald Choa Neuroscience Institute, The Chinese University of Hong Kong, Hong Kong SAR, 999077 China; 3 Nexus of Rare Neurodegenerative Diseases, The Chinese University of Hong Kong, Hong Kong SAR, 999077 China; 4 Key Laboratory of Tropical Translational Medicine of Ministry of Education, College of Biomedical Information and Engineering, Hainan Medical University, Haikou, 571199 China

**Keywords:** Microglia, Sex dimorphism, Late-onset Alzheimer’s disease, Estrogen receptor signaling, Bioinformatics

## Abstract

**Background:**

Late-onset Alzheimer’s disease (LOAD) is the most common form of dementia; it disproportionally affects women in terms of both incidence rates and severity of progression. The cellular and molecular mechanisms underlying this clinical phenomenon remain elusive and ill-defined.

**Methods:**

In-depth analyses were performed with multiple human LOAD single-nucleus transcriptome datasets to thoroughly characterize cell populations in the cerebral cortex. ROSMAP bulk human brain tissue transcriptome and DNA methylome datasets were also included for validation. Detailed assessments of microglial cell subpopulations and their relevance to sex-biased changes at the tissue level were performed. Clinical trait associations, cell evolutionary trajectories, and transcription regulon analyses were conducted.

**Results:**

The relative numbers of functionally defective microglia were aberrantly increased uniquely among affected females. Substratification of the microglia into different subtypes according to their transcriptomic signatures identified a group of female-enriched and disease-associated microglia (FDAMic), the numbers of which were positively associated with disease severity. Phenotypically, these cells exhibit transcriptomic signatures that support active proliferation, MHC class II autoantigen presentation and amyloid-β binding, but they are also likely defective in phagocytosis. FDAMic are likely evolved from female activated response microglia (ARMic) with an *APOE4* background and compromised estrogen receptor (ER) signaling that is deemed to be active among most subtypes of microglia.

**Conclusion:**

This study offered important insights at both the cellular and molecular levels into how ER signaling affects microglial heterogeneity and function. FDAMic are associated with more advanced pathologies and severe trends of cognitive decline. Their emergence could, at least in part, explain the phenomenon of greater penetrance of the *APOE4* genotype found in females. The biases of FDAMic emergence toward female sex and *APOE4 s*tatus may also explain why hormone replacement therapy is more effective in *APOE4* carriers. The pathologic nature of FDAMic suggests that selective modulations of these cells may help to regain brain neuroimmune homeostasis, serving as a new target for future drug development.

**Supplementary Information:**

The online version contains supplementary material available at 10.1186/s12974-023-02987-4.

## Background

According to the latest key facts from the World Health Organization, dementia is currently the seventh leading cause of death and one of the major drivers of disability and dependency among elderly people (World Health Organization, Dementia key facts 2023). Late-onset Alzheimer’s disease (LOAD) is the most common form of dementia, a disease that disproportionally affects women [1]. Despite these clinical observations, the underlying cellular and molecular mechanisms remain elusive. Age is perceived as the most influential risk factor for LOAD [2]. The relatively longer life expectancies among females were previously proposed to explain the sex-biased observations in the disease [3]. However, modern studies now indicate that the longevity factor alone is insufficient to clarify this issue [4], as it fails to explain why the disease manifests at a much lower prevalence in young women than in age-matched men and why the reverse trend is found among postmenopausal women [4].

It is now believed that these observations are at least in part caused by the divergent alterations in brain structures and microenvironments that male and female subjects manifest in response to disease-causing insults [5]. Since the human immune system and function are sexually distinct [6], it is proposed that sex-biased differences in the neuroimmune response may be involved [7]. Microglia are the resident innate immune cells in the brain [8, 9]. In addition to exhibiting varying structures and functions in different brain regions, sex-specific transcriptomic and proteomic profiles are observed among these cells [10, 11]. On the basis of these earlier findings, microglial heterogeneity has recently gained much attention with the emergence of single cell-based technologies. Multiple studies have utilized bulk and single-cell transcriptomic data from elderly human brains to identify different microglial properties and their relationships with neuropathology. Consistent with classic AD pathologies, it has been suggested that the genetic risk of LOAD, such as *APOE* and *TREM2* status, is functionally associated with the microglial response to amyloid-β pathology (i.e., amyloid-responsive microglia) [12–14]. On the other hand, activated response microglia (ARMs) [15], which share many overlapping homeostatic response signatures with disease-associated microglia (DAMs) [16], are generally considered a part of the normal aging response, although a subpopulation of ARMs of female origin distinguished by the expression of genes involved in MHC class II presentation, tissue repair and LOAD genetic risks (*APOE, BIN1,* etc.) has been associated with progressive amyloid-β accumulation [17]. In the same way, losses in homeostatic phenotype, phagocytic activities and activation response of DAMs are also associated with neurodegeneration [16, 18] and accumulation of degenerated myelin (i.e., white matter-associated microglia [WAMs]) [15].

With these updated findings describing the heterogeneous responses conferred by different subtypes of microglia in LOAD, recent studies have aimed to elaborate the underlying transcriptomic signatures associated with these changes. It has been repeatedly reported that microglial populations related to disease progression and proinflammatory responses exhibit heightened expression of *APOE* and lipid metabolism genes [19–21], suggesting that immunometabolic pathway perturbations possibly contribute to advanced disease pathogenesis [19]. While details on the molecular mechanisms underlying the heterogeneities of microglial phenotypes within the adult human brain are emerging, less is known about how female sex may affect the relative quantities of microglial subpopulations and whether estrogen receptor (ER) signaling plays a role in shaping certain properties of microglia related to sex dimorphisms observed in LOAD pathogenesis.

To better understand whether sex-biased and disease-specific differences in microglial populations and molecular properties exist, an in-depth investigation was conducted in multiple human brain datasets. Here, we describe both established and previously unrecognized female-enriched and disease-associated microglia (FDAMic), the evolutionary trajectory relationship among them, and the ER signaling gene network related to the observed transcriptomic changes in FDAMic. We identified that the relative populations of FDAMic are more prevalent in female LOAD patients with an *APOE4* background. This study therefore offered important insights at both the cellular and molecular levels into how ER signaling affects microglial heterogeneity and function. In addition, the biases of FDAMic emergence toward female sex and *APOE4 s*tatus may explain how hormone replacement therapy is more effective among *APOE4* carriers.

## Methods

### Single-nucleus RNA sequencing data sources, data processing and original codes

All analyses were carried out using freely available software packages. All original codes for each figure can be found at https://github.com/KimChow-Lab/FDAMic.

The following datasets were used: the Mathys et al. single-nucleus RNA sequencing (syn18485175) dataset was downloaded from Synapse.org [22] for use as the discovery cohort. A total of 70,634 high-quantity cells were input into the Seurat 3 pipeline. The first 30 principal components were considered for UMAP visualization and cell-type identification. According to known cell-type labeling and cell type-specific markers, eight major cell types were identified. Twenty-two subcell clusters were obtained at a resolution setting = 0.5. Cluster 22 (one group of excitatory neurons containing 146 cells) was removed as all the nuclei originated from one sample.

To validate the existence of FDAMic, analyses were reperformed and validated with the Morabito et al. (GSE174367) single-nucleus RNA sequencing dataset, which was downloaded from the GEO database [23]. Protein-coding genes were used to identify brain cell types, as well as markers used by the original publication [23]. Microglial populations were extracted, and batch effects between samples were removed using the align_cds function in Monocle 3 [24].

### Pseudotime cell differentiation status analysis

The expression matrix of microglia was constructed by the GetAssayData function in Seurat [25]. The Monocle 3 [26] package was used to generate pseudotime evolutionary trajectories according to the microglia expression matrix. The first 20 principal components were used to normalize the data with the preprocess_cds function, and the resolution in the cluster_cells function was set to 0.01 for clustering the cells. Microglial nuclei were ordered by learn_graph and order_cells functions. To identify the roots of the trajectory lines, the signaling entropy for each cluster was computed by the SCENT R package [27] to estimate the differentiation potential. In parallel, the CytoTRACE webserver [28] was used to validate the initial findings. Differentially expressed genes (DEGs) in different subcell clusters in microglia were identified by the FindMarkers function in Seurat with pct.min = 0.25 using the default Wilcoxon rank-sum test. DEGs with a *p* value < 0.01 were considered significantly changed genes in different subclusters of microglia.

### Identification of transcription regulons and their activities by the SCENIC algorithm

The SCENIC algorithm was developed to assess the regulatory network analysis regarding transcription factors and discovery regulons (that is, transcription factors and their target genes) in an individual cell [29]. The default databases hg19-500 bp-upstream-7species.mc9nr.feather and hg19-tss-centered-10 kb-7species.mc9nr.feather were used to analyze transcription factor binding motifs of target genes. After calculating the coexpression relationships between transcription factors and target genes in each single cell, regulons were identified by coexpression and binding location information. Estimation of the activated status of each transcription regulon for each microglial subtype was subsequently performed. Regulon activity was then analyzed by AUCell software, and any transcription factors with area under the curve (AUC) values greater than the 0.05 threshold in any of the cell clusters were retained for further analysis.

### Deconvolution of ROSMAP bulk RNA-seq datasets

The Scaden [30] and CIBERSORTx [31] algorithms were used to deconvolute the bulk RNAseq datasets from the ROSMAP study accessed from the AD knowledge portal. To optimize the program-running burden to the system, random sampling of 5000 nuclei from the Mathys et al. dataset [22] and subsequent validation on all cell type coverage were performed before being considered as the reference matrix. This read count matrix was then used to deconvolute the normalized bulk RNA-seq data by CIBERSORTx. S-mode was then selected to remove any potential batch effects. The CPM value for each cell was subsequently used to generate the training data for Scaden.

### Statistical methods for cell number variation and DEG analysis for bulk RNA-seq

The significance of cell number variations between ND and LOAD samples of different sexes was estimated by the Chi-square test. The significance of microglial cell proportions that emerged under different pathological conditions was calculated by the two-sample Wilcoxon test. Significances of differentially expressed genes between ND and LOAD samples of the ROSMAP bulk RNA-seq dataset were obtained by the linear regression model for each gene, which was implemented in the limma package by presetting the postmortem interval and age as covariate factors. Genes with *p* values less than 0.01 were regarded as significantly changed. Cell type-specific markers from single-nucleus RNA-seq datasets were identified using FindMarkers in Seurat with pct.min set at 0.25. Only DEGs with *p* values less than 0.01 and expression in more than 20% of the cells in the corresponding subcluster were considered significant. GSEA was used to compare the pathway variations among different clusters.

### Overrepresentation enrichment analysis

Overrepresentation enrichment analysis for the target gene sets was conducted on Metascape [32] using the default settings of the platform. The 20 most significantly enriched terms were visualized and analyzed as a network. The ClusterProfiler [33] package was used to compare pathways enriched between various microglial subclusters. Gene set enrichment analysis (GSEA) was used to perform a global KEGG pathway comparison among microglial subtypes. The msigdbr package was used to extract the KEGG pathways from the MSigDB database [34]. The Wilcoxon test was performed by the wilcoxauc package to prerank the genes, which was then used as the input for the fgsea package. Pathways with *p* values less than 0.01 were selected for subsequent analyses.

### Characterization of different microglial subtypes

To characterize all possible microglial subtypes identified by our trajectory analysis, gene expression signatures of these cells were compared with those identified in previous studies, as elaborated in the Results section. Genes with elevated expression in specific subgroups were selected as potential signature genes. With the fgsea R package [35], signature genes were mapped to different microglial cell clusters according to their expression distribution. Normalized enriched scores (NESs) were used to compare the relationship between paired microglial subtypes.

### Identification of estrogen-responsive methylomic gene loci and their changes in LOAD

The brain DNA methylation data of ND and LOAD patients from the ROSMAP study were downloaded from the synapse database (syn3157275). For the identification of estrogen-responsive methylomic loci, DNA methylation data of MCF-7 cells subjected to estrogen-depleted and replete conditions were used, and the dataset was downloaded from the GEO database (GSE132513). The differentiated methylated loci were identified by the limma package with the default setting. Changes at loci with *p* values less than 0.01 were considered significant.

## Results

### Microglia are aberrantly expanded but functionally compromised in female LOAD patients

**Fig. 1:**
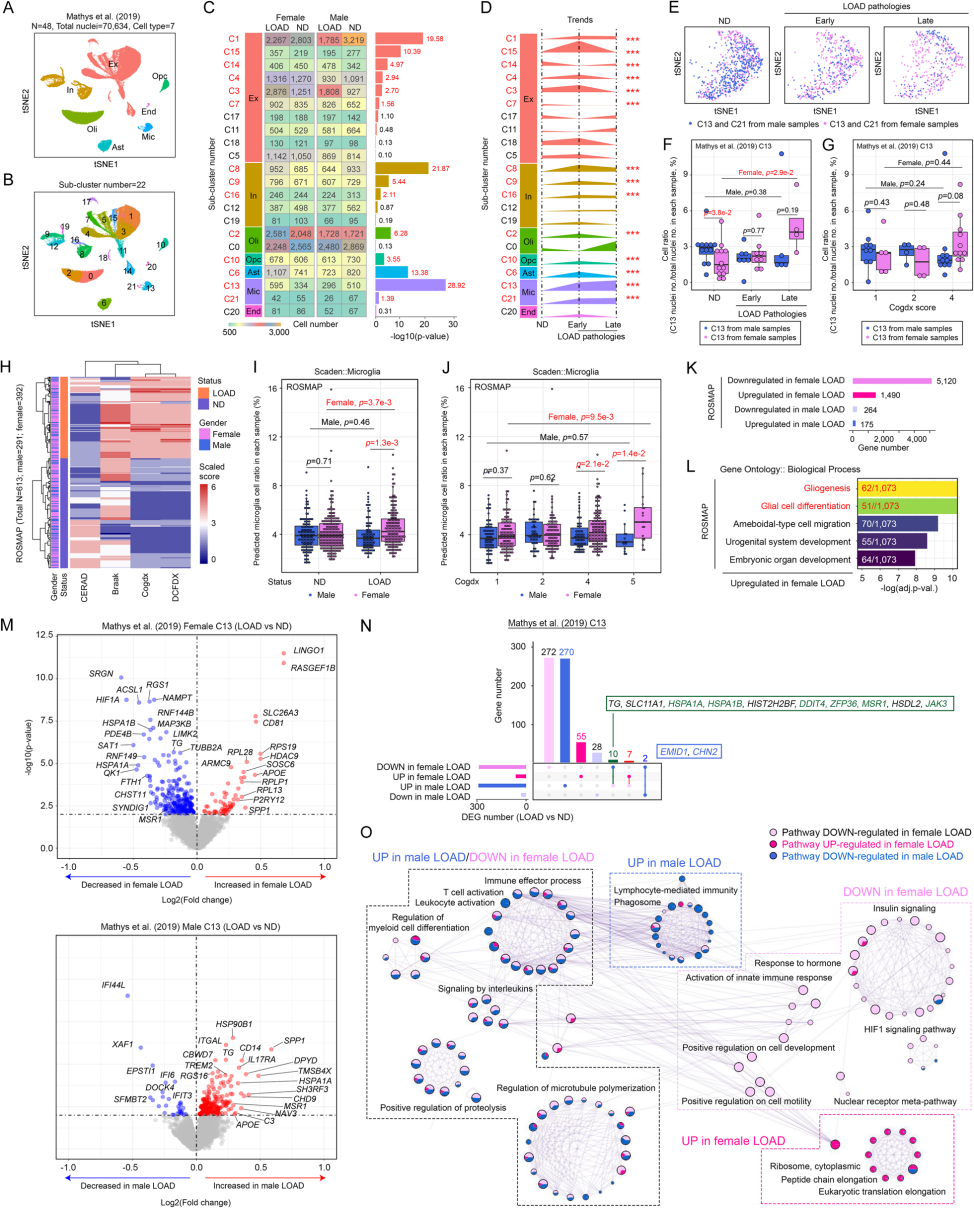
Aberrant expansion of functionally compromised microglial populations in female subjects with LOAD. **A** T-SNE plot of 70,634 nuclei derived from 48 age- and sex-matched prefrontal cortex samples harvested from both nondementia [*N* = 24 (male *N* = 12; female *N* = 12)] and LOAD [*N* = 24 (male *N* = 12; female *N* = 12)] patients from the Mathys et al. dataset. Different cell types are abbreviated as follows: excitatory neurons, Ex; oligodendrocytes, Oli; inhibitory neurons, In; astrocytes, Ast; microglia, Mic; endothelial cells, En; and oligodendrocyte progenitor cells, Opc. **B** Cells were further subclustered and colored based on subcluster numbering. **C** Cell number distribution statistics according to sex and disease status within different subclusters. Bars on the right represent − log10(*p* value). **D** Trends of changes in the scaled female:male cell ratio against disease status. ***P < 0.05. **E** T-SNE plots showing how female and male nuclei from Clusters 13 and 21 are distributed in different stages of the disease. **F**, **G** Relative cell ratio of Cluster 13 in different **F** stages of disease or **G** status of cognitive functions (Cogdx scores). **H** Disease status of samples of the ROSMAP study was defined based on multiple clinicopathological parameters (*x*-axis). **I**, **J** Relative microglial cell ratio changes in ROSMAP samples calculated by the Scaden deep learning algorithm. Comparisons were made between various **I** disease statuses and **J** stages of cognitive function (Cogdx scores). **K** Number of DEGs identified from sex-specific comparisons made between LOAD and ND samples of the ROSMAP dataset. **L** Gene Ontology: biological process pathway enrichment analysis of downregulated DEGs in female LOAD samples. **M** Volcano plots illustrating DEGs curated from the comparison between Cluster 13 nuclei originating from LOAD versus ND samples in a sex-specific manner [females (top panel) and males (bottom panel)]. **N** UpSet plot illustrating how DEGs shown in **M** overlapped and were related to one another. **O.** Metascape enrichment network of DEGs shown in **M** and corresponding boxes indicating how different pathways manifested in LOAD samples of different sexes. Pathways that shared similar trends of changes in a particular sex are grouped together in dashed boxes. Color code: light pink (downregulated pathways in female LOAD); dark pink (upregulated pathways in female LOAD); dark blue (upregulated pathways in male LOAD)

Previous studies performed in laboratory mouse models showed that altered microglial physiology contributes to the sex-dimorphic effects observed in LOAD [17, 36–38]. To better understand their relevance to humans, pilot data analysis of a single-nucleus RNA-sequencing (snRNA-seq) dataset from Mathys et al. [22] consisting of 48 age- and sex-matched prefrontal cortex samples harvested from both nondementia [*N* = 24 (male *N* = 12; female *N* = 12)] and LOAD [*N* = 24 (male *N* = 12; female *N* = 12)] individuals was performed. From a total of 7 major cell types (Fig. 1A, Additional file 10: Fig. S1A), 22 smaller clusters were substratified based on refined analysis of their transcription profiles (Fig. 1B). Subsequent analyses of the cell number distribution with respect to sex and disease status revealed significant differences in 14 out of 22 subclusters (Fig. 1C). Among them, Cluster 13 was the most significant, characterized by an enriched population of female microglial nuclei from affected individuals (Fig. 1C). The existence of this female-enriched and disease-associated group of microglia was confirmed in another dataset deposited by Lau et al., although the finding was not statistically significant due to the small sample size [39] (Additional file 10: Fig. S1B, C). With reference to an existing definition of disease severity in the original publication [22], which was preassigned based on an integrated consideration of multiple clinicopathological features (Additional file 1: Table S1), changes in total female-to-male cell number ratios between nondementia (ND) and disease-affected (LOAD) samples were investigated. No obvious sex biases were found at the whole-tissue level (Additional file 10: Fig. S1D); significant differences were found within 14 subclusters of cells (Fig. 1D). Of all possible brain cell types detected, microglia were the only ones that consistently exhibited a progressive increase in cell number as the disease advanced in females (Fig. 1D). Relative to nondementia controls, the increment in microglial cell number observed in female samples was more profound at advanced stages of the disease (Fig. 1E, F) and was positively associated with more severe neurofibrillary tangle (NFT) deposition (i.e., defined by Braak and Cerad scores) (Additional file 10: Fig. S1E, F). Despite being marginally insignificant, the relative quantities of microglia also trended upward in female subjects suffering from more severe cognitive decline (i.e., Cogdx score) (Fig. 1G).

To further validate these relationships, additional analyses in the bulk transcriptomic datasets of brain tissues harvested from 613 individuals (nondementia: male *N* = 133 and female *N* = 219; LOAD: *N* = 88 and female *N* = 173) that took part in the Religious Orders Study and Memory and Aging Project (ROSMAP) [40] were performed. Similarly, the phenotypic status of these samples (i.e., LOAD vs. ND) was defined with reference to multiple clinicopathological features, including the neuritic plaque load (CERAD) [41], neurofibrillary tangle pathology (Braak) [42] and cognitive status [Cogdx [43] and DCFDX [44]] (Fig. 1H). Then, cell composition analysis was performed with the deep learning-based Scaden method [30], which revealed selective increments in relative microglial cell number among affected females (Fig. 1I, J); this finding was alternatively validated by the CIBERSORTx deconvolution method [31, 45] (Additional file 10: Fig. S1G, top). Further analyses also indicated that these observations were associated with a more advanced disease status, as reflected by poorer Cogdx (Fig. 1J), Braak and Cerad scores (Additional file 10: Fig. S1G).

The observed increase in microglial cell number suggested that the global homeostatic status of the cells may have changed. In the ROSMAP bulk transcriptomic dataset, DEG analysis between sex-specific LOAD versus ND samples revealed a significantly higher number of DEGs in female than male samples (DEG_LOAD vs nondementia_: female samples = 4643 genes; male samples = 446 genes) (Fig. 1K, Additional file 2: Table S2). Among these, upregulated DEGs in the female LOAD group were clustered significantly into pathways related to gliogenesis and glial cell differentiation (Fig. 1L), suggesting that these cells are possibly evolving to a more terminally differentiated state or adopting a more stable reactive phenotype. Notably, this sex-biased trend in the number of DEGs and the functional implication of upregulated DEGs in female LOAD patients were validated even after downsizing the female sample number to be equal to that of males, as revealed by random elimination of female samples 1000 times in a permutation analysis (Additional file 2: Table S2). This finding validated that the DEGs identified were not biased by the preexisting differences in sample numbers. Cell-specific DEG analyses performed in sex-specific LOAD versus ND samples of Mathys et al.’s dataset (Fig. 1M) revealed that key microglia-related proliferation genes, such as *CSF1R* [46] and *CD81* [47], were significantly induced in affected females (Additional file 10: Fig. S1H-I). Further analysis of the DEGs that were downregulated in affected females but upregulated in affected males was performed (Fig. 1N). Genes related to microglial stress responses, such as *HSPA1A* and *HSPA1B*, which prevent protein aggregation [48]; *DDIT4*, which modulates Aβ cytotoxicity [49]; *ZFP36*, which downregulates proinflammatory cytokine production [50]; and *MSR1*, which supports alternatively activated (M2) polarization of macrophages [51], were identified (Fig. 1N). In contrast, the DEGs that manifested in an opposite manner (i.e., downregulated in affected females but upregulated in affected males) included *EMID1* and *CHN2*, which promote cell proliferation and migration [52, 53] (Fig. 1N). These findings suggested that enhanced cell proliferation but diminished stress response signaling networks may preferentially occur in the microglia of affected females. This assumption was supported by the ROSMAP dataset, as immune function pathways, such as T cell activation, interleukin signaling and positive proteolysis regulation, were consistently downregulated uniquely in the affected female group (Fig. 1O). Similarly, these sex-biased changes at the pathway level were also found in Cluster 13 (i.e., microglia) of the Mathys et al. dataset (Additional file 10: Fig. S1J, left panel). Notably, these observations were readily evident even in the early stages of the disease (Additional file 10: Fig. S1J, right panel). Collectively, these data confirmed that transcriptomic changes in microglia in LOAD patients were sexually biased.

### Identification of female-enriched and disease-associated microglia (FDAMic)

**Fig. 2:**
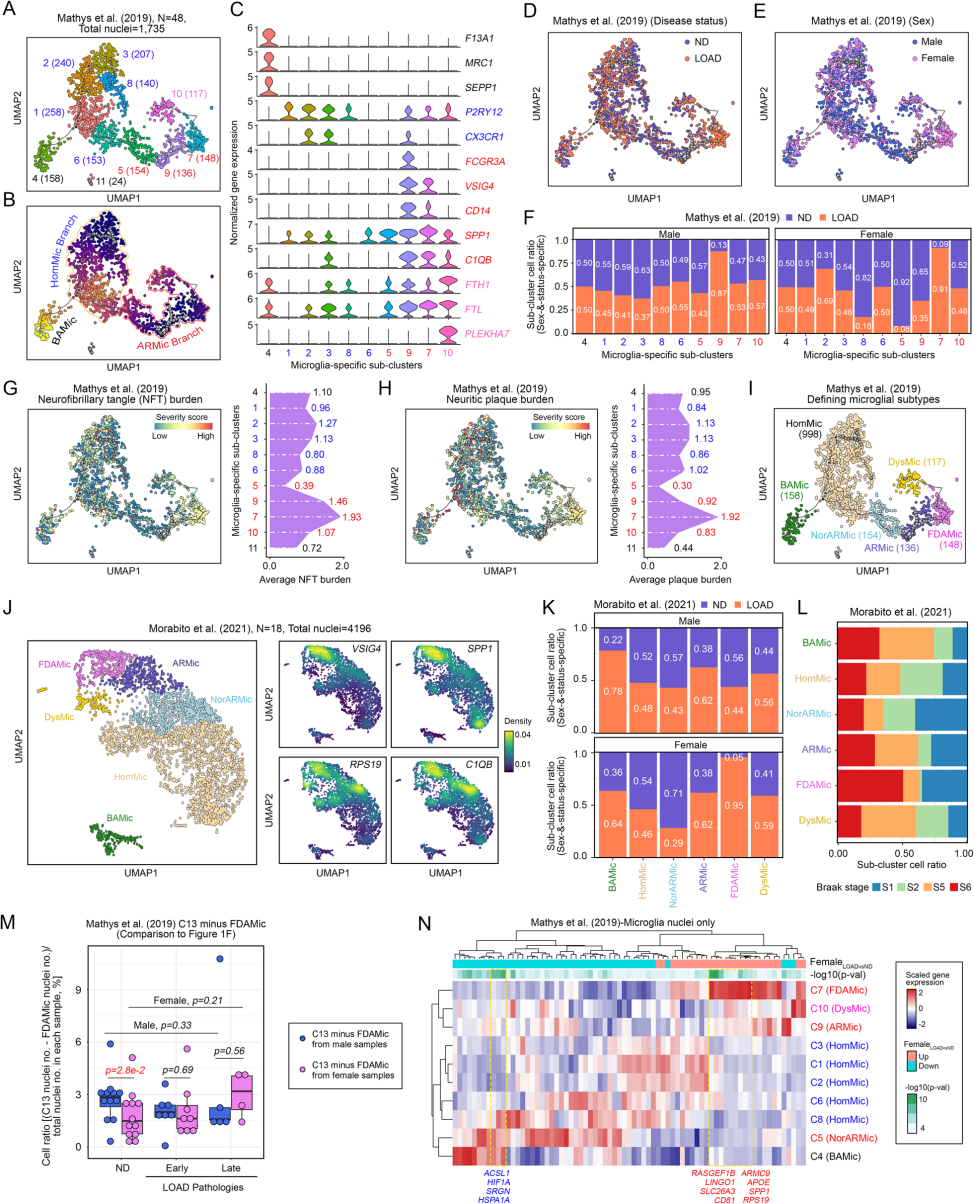
Identification of female-enriched and disease-associated microglia (FDAMic). **A**, **B** Single-cell trajectory analysis with the Monocle 3 algorithm identified an evolutionary relationship among all microglial nuclei in the Mathys et al. cohort. **A** UMAP plot showing the locations of various microglial subclusters based on existing knowledge from published studies. Numbers inside the blanket represent the number of nuclei. **B** Three major branches of microglial fates were identified: HomMic, BAMic and ARMic branches. **C** Violin plots showing marker gene expression of BAMic (black), HomMic (blue), ARMic (red) and DysMic (pink) subtypes. **D**, **E** Visualization of all subtypes of microglia on the evolutionary trajectory UMAP plot according to **D** disease diagnosis or **E** sex of the samples. **F** Sex- and disease status-specific cell ratio distribution among all subclusters of microglia. Subcluster numbers are color-labeled according to their branch location on the evolutionary plot, i.e., BAMic (black), HomMic (blue), and ARMic (red). **G**, **H** Visualization of all subtypes of microglia on the evolutionary trajectory UMAP plot according to **G** neurofibrillary tangle (NFT) burden or **H** neuritic plaque burden of the samples. **I.** Detailed definition of microglial subtypes defined by our analysis, particularly the female-enriched and disease-associated microglia (FDAMic). **J** Single-cell trajectory analysis with the Monocle 3 algorithm identified an evolutionary relationship among all microglia in the Morabito et al. cohort. The expression intensities of the *VSIG4, SPP1, RPS19* and *C1QB* genes are indicated on the right. **K** Sex- and status-specific cell ratio distribution among all subclusters of microglia identified in the Marabito et al. study. **L** Ratios of nuclei in samples of different Braak stages belonging to different subclusters of the Marabito et al. cohort. **M** Relative cell ratio changes in Cluster 13 of the Mathys et al. cohort after selective removal of FDAMic nuclei from the analysis. **N** Relative expression levels of the DEGs curated from the comparison between Cluster 13 nuclei originating from female LOAD versus ND samples (Fig. 1M, top panel) in different subtypes of microglia of the Mathys et al. cohort

To delineate whether the expansion of functionally compromised microglia in affected females is a general phenomenon occurring in the majority of microglia cells or in a distinct subgroup of microglia [54, 55], a semisupervised pseudotime analysis was performed based on the microglial differentiation status [26] (Fig. 2A, Additional file 11: Fig. S2A). From the analysis, three major trajectory branches were defined. Subcluster 11 was completely disconnected from the others, and due to the small cell number, it was discarded in the subsequent analyses (Fig. 2A, Additional file 11: Fig. S2A). In this setting, the “root” of the trajectory, defined as the most undifferentiated state [28], was identified based on the assumption that undifferentiated cells process more diverse gene expression profiles, while terminally differentiated cells are highly specialized [28]. Using the SCENT entropy-based method, cells labeled with higher entropies representing greater gene expression profile diversity were first deployed in the analyses [27, 56, 57], which revealed that Subclusters 3 and 9 (Fig. 2A) were likely the roots of the two separated branches (Additional file 11: Fig. S2B, top panel). This observation was alternatively validated by cytoTRACE, an algorithm that empirically uses the number of expressed genes per cell as a measure of transcription diversity (Additional file 11: Fig. S2B, bottom panel) [28]. To better understand the degree of cell differentiation and functional status of microglia located along the trajectory branches, mapping analysis for known marker genes was performed. The branch that consisted of Subclusters 1, 2, 3, 6 and 8 (Fig. 2A, B) exhibited enriched gene expression of classic homeostatic microglial (HomMic) markers, such as *P2RY12* and *CX3CR1* (Fig. 2C, Additional file 11: Fig. S2C), and was therefore considered the “HomMic Branch”. The branch constituted by Subclusters 5, 7, 9 and 10 exhibited a much more diverse set of microglial signatures (Fig. 2C, Additional file 11: Fig. S2C). For instance, Subcluster 10, located at the terminus, resembled disease-associated dystrophic microglia (DysMic) [14] due to the robust expression of *FTL1, FTH* and *PLEKHA7* (Fig. 2C, Additional file 11: Fig. S2C). In the neighboring Subclusters 5, 7 and 9, however, robust expression levels of activated response microglia (ARMic) markers, such as *SPP1* and *C1QB,* were observed (Fig. 2C, Additional file 11: Fig. S2C). Since Subcluster 9 was predicted as a “root” (Fig. 2A, Additional file 11: Fig. S2B), this finding matched the established role of ARMic as a precursor of DysMic [17]. Subcluster 4, which dominated a distinct branch (Fig. 2A-B), resembled border-associated macrophage-like microglia (BAMic) due to its robust expression of *F13A1*, *MRC1* and *SEPP1* (Fig. 2C, Additional file 11: Fig. S2C) [58].

Using these subtype classifications, we then further analyzed the relationship with disease status (Fig. 2D) and sex (Fig. 2E) distributions in each subcluster. Notably, Subcluster 7 was highly enriched with microglia from multiple affected female samples (Fig. 2F, Additional file 11: Fig. S2F). Further characterization of the subcluster relationship to the severity of disease in terms of neurofibrillary tangle (NFT) load (Fig. 2G), neuritic plaque burden (Fig. 2H), and severity of cognitive impairment (Additional file 11: Fig. S2D) supported that Subcluster 7 was associated with a more advanced disease status in the absence of biases of age (Additional file 11: Fig. S2E) or donor effect (Additional file 11: Fig. S2F). Collectively, Subcluster 7 was defined as the “female-enriched and disease-associated microglia” (FDAMic) cluster (Fig. 2I), and their emergence was validated further in another cohort of samples [23] (Fig. S2G, validation cohort). Consistently, FDAMic was located between ARMic and DysMic on the pseudotime trajectory (Fig. 2J, Additional file 11: Fig. S2H). Moreover, this cluster of cells identified in the validation cohort was enriched with nuclei from affected female subjects (Fig. 2K) with a more advanced Braak status (Fig. 2L).

To elaborate the contribution of FDAMic to the global molecular changes detected in all microglia, cell nuclei belonging to this cluster were selected from the entire discovery cohort. The positive relationship between relative C13 cell number and disease severity was no longer significant (Fig. 1F versus 2 M). Similar to the list of DEGs curated from comparing C13 cells of female LOAD versus ND samples (Fig. 1M), a substantial number of upregulated DEGs were contributed uniquely by FDAMic (Fig. 2N, Additional file 11: Fig. S2I). Next, upon selective elimination of FDAMic nuclei, the statistical significance and fold changes of many DEGs were diminished (Additional file 11: Fig. S2J). These included the microglia-specific neuroinflammatory-stable gene *CD81* [59]; major brain cholesterol carrier *APOE* [60], *SPP1*, which mediates phagocytic activities [61]; and immunosuppressive ribosomal protein S19 (*RPS19*) [62] (Additional file 11: Fig. S2J, K). Notably, these changes were not observed when an equal number of random microglial nuclei were eliminated in the control permutation analyses (Additional file 3: Table S3), which alternatively validated the contribution of FDAMic to the detected global molecular changes.

### Transcriptomic signatures suggested a proliferative and proinflammatory but defective phagocytic phenotype in FDAMic

**Fig. 3:**
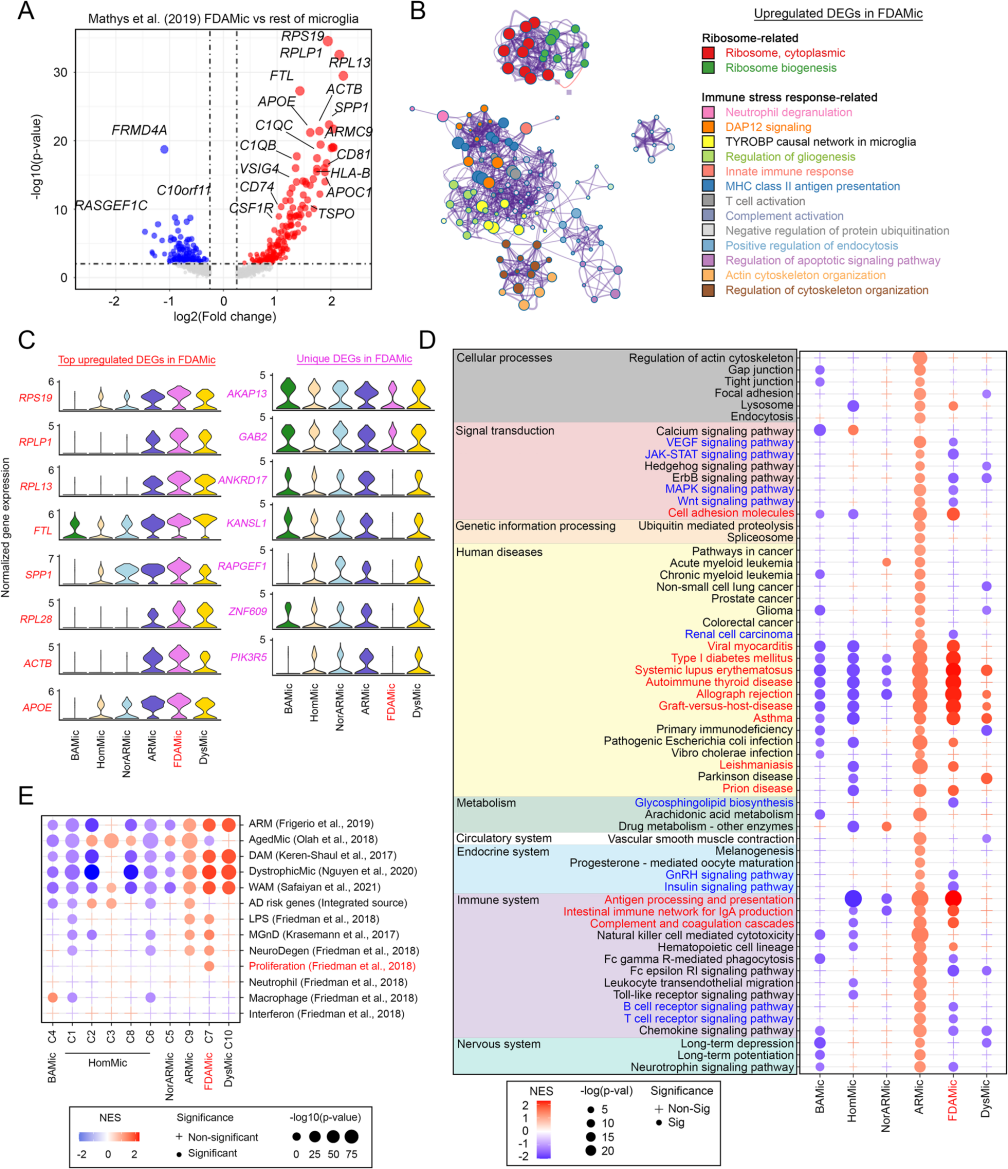
FDAMic are unique from the rest of the microglial population. **A** Volcano plot illustrating DEGs in FDAMic versus the rest of the microglial population in the Mathys et al. cohort. **B** Over‐representation analysis (ORA) of upregulated DEGs in FDAMic shown in **A** using the Metascape platform. Every node represents an enriched term, and two nodes are linked if their Kappa similarities are higher than 0.3. Similar functional terms are clustered together and are displayed using the same color. Node size is proportional to the number of enriched genes. **C** Left: Violin plots of the top 8 upregulated DEGs specific to FDAMic. Right: Violin plots of 7 downregulated DEGs specific to FDAMic. **D**, **E** Pathway analysis of DEGs in each subtype of microglia (i.e., obtained when each specific subtype was compared to the rest of the microglial populations) referencing **D** the KEGG pathway database or **E** the signature of microglial subtypes defined by different research groups as indicated

To further characterize the phenotypic properties of FDAMic in comparison to other subtypes of microglia, DEG analysis was first performed regardless of the sex or disease status of the samples. In the Mathys et al. discovery cohort, 373 DEGs were found, 183 of which were significantly upregulated in FDAMic (Fig. 3A, Additional file 4: Table S4). Subsequently, Metascape pathway and network-level analyses [32] revealed that the majority of these upregulated genes supported a network of TYROBP/DAP12-complement signaling pathways related to LOAD pathogenesis [63] (Fig. 3B). Furthermore, a group of genes encoding the major histocompatibility complex (MHC) class II autoantigens was identified, suggesting that FDAMic are likely proinflammatory in nature and may actively interact with brain-infiltrated peripheral T cells [64] (Fig. 3B). In addition to these downstream immune functions, upstream pathways such as ribosome biogenesis that support cell proliferation [65], as well as the pathway that negatively regulates protein ubiquitination [66], were identified (Fig. 3B). In contrast, the 190 downregulated DEGs in FDAMic were implicated mainly in the Rac and Rho GTPase signaling network (i.e., “RAC GTPase cycle”, “RHO GTPase cycle” and “Activation of GTPase activity” pathways) and cellular phagocytosis-related activities (i.e., “Fc gamma receptor dependent phagocytosis”, “Rc gamma R-mediated phagocytosis”, “Phagocytosis” and “Ubiquitin-dependent protein catabolic process”) (Additional file 12: Fig. S3A). Together, these findings suggested that FDAMic are likely pathological and dysfunctional. At the individual gene level, the top upregulated set of DEGs identified (Fig. 3A) (i.e., *RPS19, RPLP1, RPL13, FTL, SPP1*, *RPL28, ACTB* and *APOE*) was also robustly expressed in the ARMic and DysMic clusters (Fig. 3C), supporting their interrelationships in the ARMic branch (Fig. 3C). The only differences among the three subtypes were found in several downregulated DEGs, including *AKAP13, GAB2, ANKRD17, KANSL1, RAPGEF1, ZNF609* and *PIK3R6*, which were robustly expressed in ARMic and DysMic but were distinctively suppressed in FDAMic (Fig. 3C, Additional file 12: Fig. S3B, C).

To better characterize and visualize the functional similarities and differences between FDAMic and other subtypes of microglia in a more holistic manner, KEGG pathway analyses were performed. ARMic, as the name implies, exhibited a robust, activated signaling profile (Fig. 3D). In contrast, BAMic, HomMic and NorARMic (i.e., a subgroup of ARMic enriched with nuclei originating from nondementia samples) presented a generally inactive signaling profile (Fig. 3D). FDAMic, however, resembled a mixed functional profile. Pathways representing a set of autoimmune diseases (i.e., viral myocarditis, type I diabetes mellitus, systemic lupus erythematosus, autoimmune thyroid disease, allograph rejection, graph-versus-host-disease, asthma, leishmaniasis and prion diseases) and disease-associated immune signatures (i.e., antigen processing and presentation, intestinal immune network for IgA production and complement and coagulation cascades) were activated with MHC class II autoantigens as common lead genes (Fig. 3D, Additional file 12: Fig. S3D). Among the inactivated pathways (i.e., those highlighted in blue), common genes with a GO biological function belonging to Fc receptor signaling were suppressed and were versatilely involved in Wnt (Additional file 12: Fig. S3E), VEGF (Additional file 12: Fig. S3F) and MAPK (Additional file 12: Fig. S3G) signaling networks, as well as the T cell and B cell receptor-mediated pathways (Additional file 12: Fig. S3H-I) and microglial gonadotropin-releasing hormone (GnRH) signaling, a hypothalamic hormonal pathway that mediates reproductive [67] and metabolic competences [68] (Additional file 12: Fig. S3J). In addition, JAK-STAT (Additional file 12: Fig. S3K), insulin (Additional file 12: Fig. S3L), and glycosphingolipid biosynthesis (Additional file 12: Fig. S3M) pathways were also suppressed (Fig. 3D). To further validate the uniqueness of FDAMic from other subclusters more systematically, comparisons to signature gene expression profiles of microglial subtypes defined previously by different research groups were performed (Fig. 3E). Similar to DysMic (C10), FDAMic also closely resembled “ARM” as defined by Frigerio et al. [17]; “DAM” as defined by Keren-Shaul et al. [16]; “dystrophic microglia” as defined by Nguyen et al. [14]; and “WAM” as defined by Safaiyan et al. [15]. However, unlike DysMic, FDAMic also presented properties of the “microglial neurodegenerative phenotype” (MGnD) defined by Krasemann et al. [69], such as “neurodegenerative disease” (NeuroDegen) and “proliferation” phenotypes defined by Friedman et al. [70], which differentiated the two subtypes. In the comparison between FDAMic (C7) and ARMic (C9), the latter exhibited robust similarities to the “aged microglia phenotype” (AgedMic) defined by Olah et al. [71] but not the “proliferation” properties defined by Friedman et al. [70], which therefore differentiated the two subtypes. Collectively, these cell characterization data confirmed that FDAMic is unique from other microglial subtypes. The emergence of FDAMic is associated with sex dimorphism and the pathogenesis of LOAD.

### FDAMic evolved from female ARMic associated with a compromised estrogen receptor signaling network

**Fig. 4:**
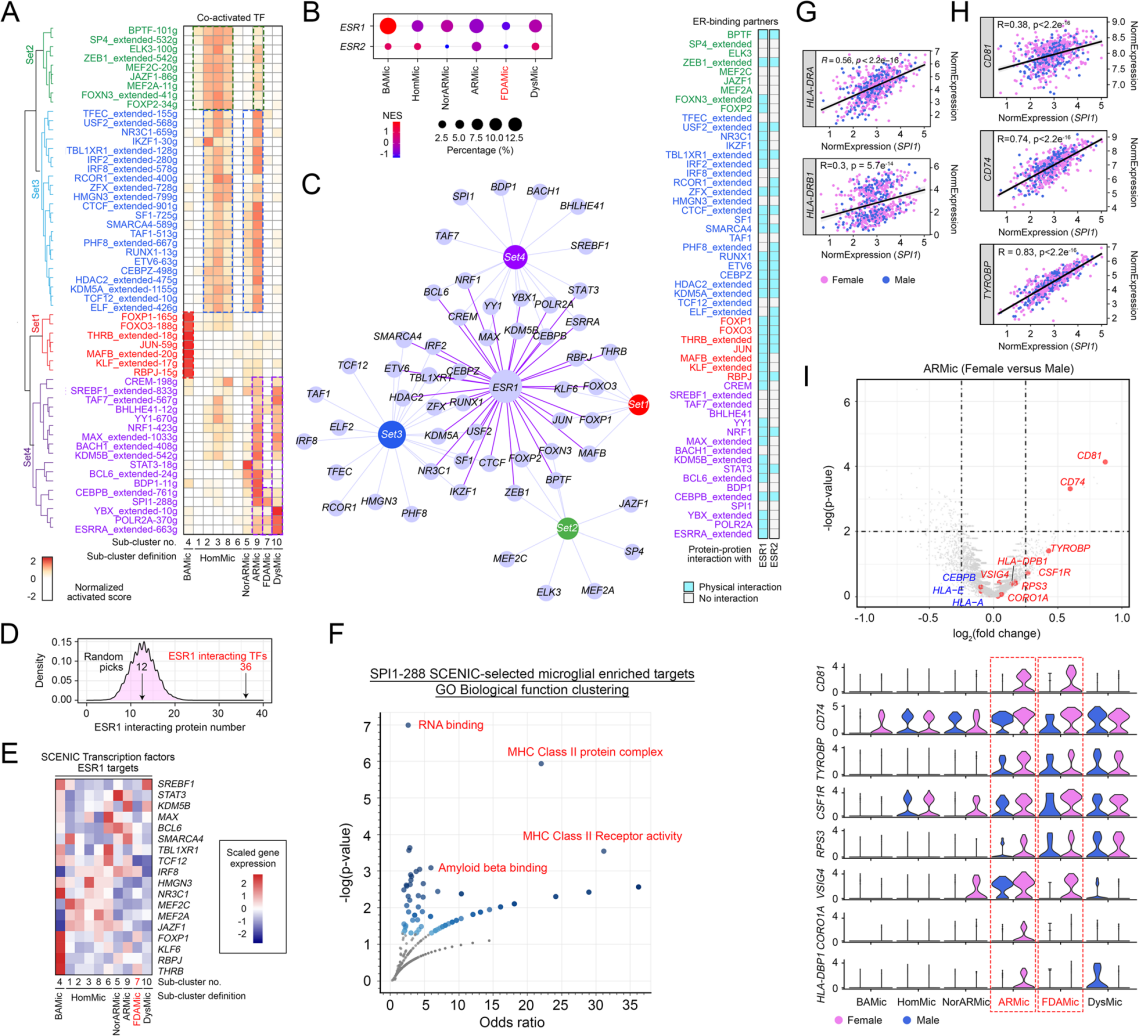
FDAMic evolved from female ARMic associated with a compromised estrogen receptor signaling network. **A** Transcription regulons that possibly regulated the DEGs in all subtypes of microglia were predicted by the SCENIC algorithm. Their degrees of activation are indicated by the color. **B** Dot plot showing the relative expression levels of *ESR1* and *ESR2* in different subtypes of microglia. **C** Left (STRING network): physical interaction network between *ESR1*/ERα and the 4 sets of transcription regulons identified by the SCENIC algorithm. Transcription factor labels indicate positive interactions. Right (heatmap): physical binding prediction between transcription factors predicted with *ESR1*/ERα or *ESR2*/ERβ. **D** Random permutation of transcription factors (TFs) and their probabilities of interacting with *ESR1*/ERα. On average, 12 out of 55 (probability = 0.22) randomly selected TFs may interact with *ESR1*/ERα; this is in stark contrast to 33 out of 55 (probability = 0.6) identified from the SCENIC algorithm. **E** Heatmap showing the scaled gene expression level of *ESR1*/ERα-targeted TFs in different subclusters of microglia. **F** Functional enrichment analysis of the 288 *SPI1* target genes highly expressed in microglia. **G**, **H** Correlations between the normalized expression level of *SPI1/*PU.1 and those of various **G** MHC class II autoantigens or **H** cell proliferation-associated genes. All of these are downstream targets of *SPI1/*PU.1*.* **I** Top: volcano plot showing DEGs identified from the comparison between ARMic of female versus male origins. Key microglial MHC autoantigens and proliferation-associated genes are labeled. Bottom: violin plots illustrating the expression profiles of these key genes across all subtypes of microglia

Our data supported that FDAMic possess a distinct set of molecular phenotypes. To identify the potential upstream drivers that shape this unique transcriptome profile, the SCENIC algorithm was deployed to dissect and compare the simultaneous gene regulatory networks among all the subtypes of microglia [29] (Fig. 4A). A set of transcriptomic regulons (i.e., Set 1) constituting FOXP1, which supports cognitive functions [72], and FOXO3 and PBPJ, which protect against age-related vascular diseases [73, 74] (Fig. 4A), was activated in BAMic. For HomMic clusters (Clusters 1, 2, 3, 6 and 8), however, alternative sets of activated transcriptomic regulons (i.e., Set 2) were activated. These proteins include BPTF [75] and MEF2C [76], which govern microglial homeostatic responses; MEF2A, which promotes autophagy [77]; and ZEB1, which mediates protective effects after brain ischemia [78] [75, 79, 80] (Fig. 4A, Set 2). Another set (i.e., Set 3) characterized by IKZF1 [81], RUNX1 [82], ETV6 [83], SMARCA4 [84], TFEC [85], RCOR1 [86] and TCF12 [87], which are all crucial for normal immune lineage commitment, was coactivated in these cells, as were NorARMic and ARMic in a different category (Fig. 4A, Set 3). In contrast, transcription regulon Sets 1–3 were not active in either FDAMic or DysMic; however, in the latter, a distinct set of transcription regulons that promote metabolic reprogramming (i.e., ESRRA [88], BACH1 [89]) and immune exhaustion (i.e., CREM [90], YBX1 [91]) was activated instead (Fig. 4A, Set 4). The FDAMic transcriptomic profile exhibited no activities in any of the 4 sets of transcriptomic regulons, suggesting that this cluster is molecularly distinct from others. One obvious distinct property of FDAMic is female nuclei enrichment (Fig. 2E, F), but they are also likely defective in gonadotropin-releasing hormone (GnRH) signaling (Additional file 12: Fig. S3J) and insulin signaling (Additional file 12: Fig. S3L) [92–94], which are known to be estrogen-regulated, suggesting that this hormonal signaling axis is key. Supporting this, estrogen receptor-1 (*ESR1*/ERα) and estrogen receptor-2 (*ESR2*/ERβ) were both the least expressed in FDAMic (Fig. 4B). One immediate consequence is the loss of protein‒protein interactions and the regulatory effects on known coactivators and transcription factors [95]. Of the 55 microglia-relevant transcription factors identified by the SCENIC program (Fig. 4A), 39 (71%) were ERα or ERβ protein-binding partners, as predicted by the STRING and PPI networks available in the BIOGRID database (Fig. 4C, Additional file 5: Table S5). This high percentage of binding partners within the list was nonrandom in nature, since this number was much higher than any random selections conducted from the entire genome (Fig. 4D). Alternatively, loss of the ER may directly downregulate its target gene expression, which includes some of the SCENIC-identified transcription factors. According to the CHEA transcription factor targets database [96], as much as 32% (18/55) of this set of microglia-relevant transcription factors were indeed the transcription targets of *ESR1*/ERα (Fig. 4E). Taken together, these findings suggested that at least a significant part of the immune-related properties shared by the common microglial subtypes except FDAMic are associated with an active ER signaling network. In other words, the aberrant emergence of FDAMic is at least in part a result of defective ER signaling, in addition to other ER-independent mechanisms that shape its phenotype.

Referring back to the SCENIC transcriptomic regulon profile, the proto-oncogene product of *SPI1* (PU.1) implicated in the pathogenesis of LOAD [97, 98] was the only activated regulon found in FDAMic (Fig. 4A), being unknown to interact with any isoform of ER (Fig. 4C) or a downstream gene target (Fig. 4E). Further pathway analysis of the 288 SCENIC-identified PU.1 targets that were highly expressed in the testing dataset suggested that they were likely involved in some of the activated features of FDAMic, including Aβ binding, MHC class II protein complex assembly and related activities (Fig. 4F, G, Additional file 6: Table S6). On the other hand, FDAMic was only a subcluster of microglia that exhibited “proliferative” properties as defined by Friedman et al. [70], which could be mediated in part by the transcriptional activities of PU.1, as it is closely associated with the expression of *CD81* [47], *CD74* [99] and *TYRPBP*/DAP12 [100] (Fig. 4H). Indeed, an activated PU.1 transcription regulon was also found in ARMic (Fig. 4A), suggesting that FDAMic could have evolved from some of these cells facing compromised activities in the ER signaling-regulated transcription network (i.e., Sets 1–4). Intriguingly, ARMic are enriched with relatively more nuclei from male subjects (Fig. 2K); therefore, it is imperative to understand whether the activated PU.1 observed in this cluster was more related to a distinct subset of nuclei derived from the female subjects. Analysis of DEGs between ARMic nuclei of female and male origin revealed that key targets of PU.1, including *CD81, CD74*, *HLA-DPB1, TYROBP* and *CSF1R* [101], were more robustly expressed in female nuclei (Fig. 4I), suggesting that this subset of cells is a potential precursor of FDAMic.

**Fig. 5:**
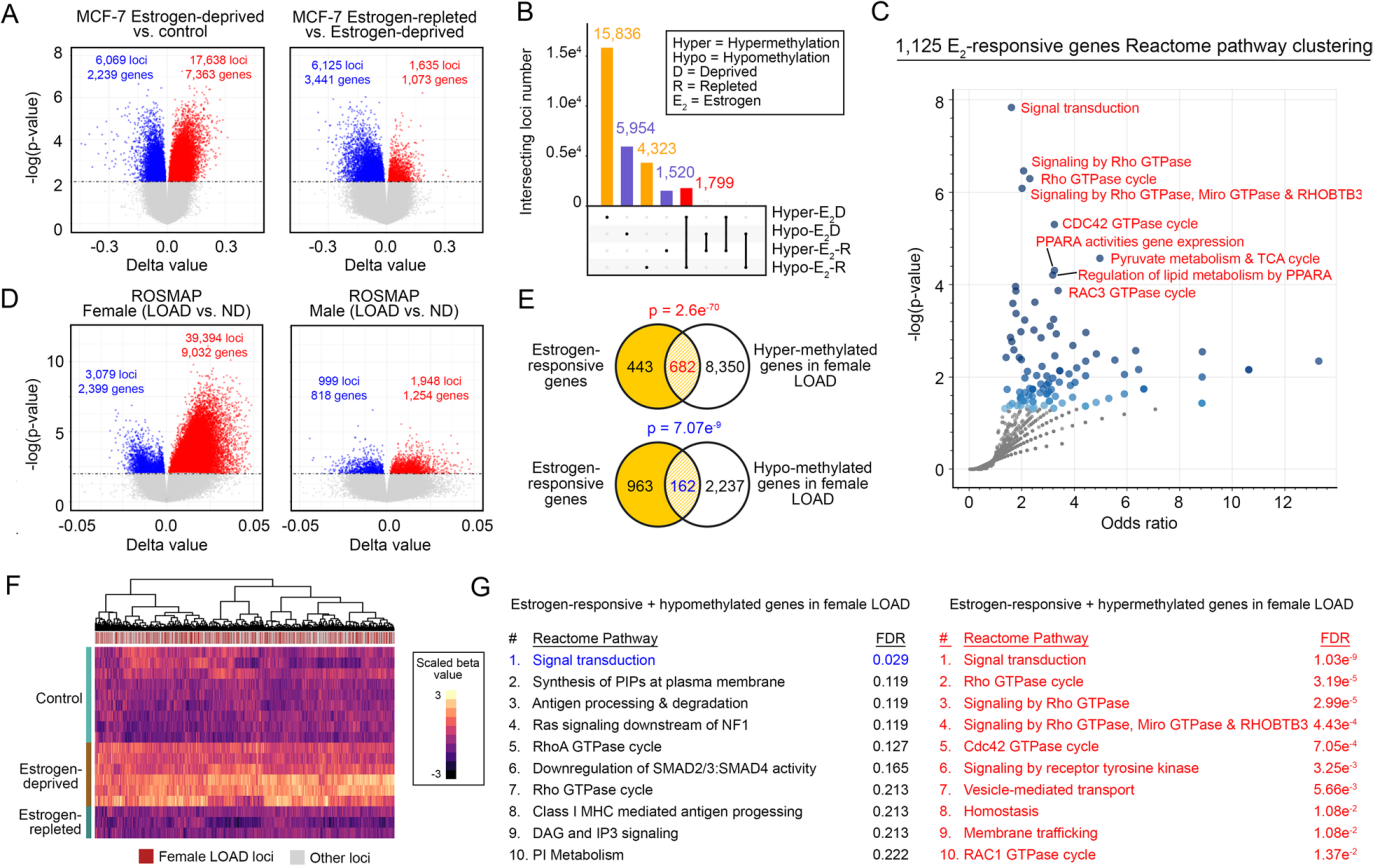
Estrogen receptor signaling regulates Rac and Rho GTPase signaling network by altering the global DNA methylation profile. **A** Volcano plots illustrating the DNA methylation profiles of the MCF-7 cell line subjected to estrogen-deprived (left) or replete (right) conditions. **B** UpSet plot summarizing the total and intersecting hyper- or hypomethylated locus numbers identified in **A**. **C** Pathway enrichment analysis of 1125 estrogen-responsive DNA methylation gene loci identified from **A**. The top enriched pathways are labeled. **D** Volcano plots showing the changes in DNA methylation profiles in brain tissues of LOAD versus ND individuals in a sex-specific manner (left: female; right: male). **E**, **F** Estrogen-responsive DNA methylation gene loci revealed significant overlap with altered DNA methylation loci found in female LOAD patient samples, illustrated as **E** Venn diagram and **F** heatmap formats. **G** Pathway enrichment analysis of estrogen-responsive hypomethylated (left) and hypermethylated (right) genes identified in female LOAD subjects

In addition to these trans-acting mechanisms, ER signaling may also epigenetically modulate DNA methylation to adjust chromatin accessibility for various transcription regulators in estrogen-sensitive cells [102]. Using the estrogen-sensitive MCF-7 DNA methylome as a testing model, depletion of the hormone in the culture environment resulted in a more pronounced gain in global DNA methylation than in demethylation (Fig. 5A, left), and these observations were reversed when the hormone was repleted back into the system (Fig. 5A, right). From these observations, 1,799 estrogen-responsive DNA methylation loci (i.e., the majority of genetic regions that become hypermethylated under estrogen depletion but respond in a reverse manner upon estrogen repletion, as well as the very few that respond the other way round) found on 1,125 genes were identified (Fig. 5B, Additional file 7: Table S7). Functionally, an ample number of these genes were intriguingly implicated in the signaling network of Rac and Rho GTPases (Fig. 5C). Considering that the regulatory regions of these genes are likely hypermethylated, their expression is likely suppressed [103] in the absence of ER signals; therefore, this epigenetic mechanism may explain how genes involved in the Rac and Rho GTPase signaling network are suppressed in FDAMic (Additional file 12: Fig. S3A). To validate whether these loci were affected in LOAD, comparisons of DNA methylome profiles between LOAD and ND brain samples were performed in a sex-specific manner. The analysis revealed that changes in DNA methylation to female LOAD subjects were more dramatic than those found in males (Fig. 5D). In female LOAD subjects, a total of 39,394 hypermethylated loci corresponded to 9,032 genes, and 3,079 hypomethylated loci corresponded to 2,399 genes (Fig. 5D, Additional file 8: Table S8). Notably, a significant number of loci were likely estrogen-responsive, as suggested by the comparative analysis (Fig. 5E) against the list curated from the MCF-7 analysis (Fig. 5A, B). From there, 162 hypomethylated estrogen-responsive genes were identified; however, they were not clustered into any meaningful pathways other than “signal transduction” (Fig. 5E–G). In contrast, among the 682 hypermethylated estrogen-responsive genes identified (Fig. 5E, F), many were implicated in multiple pathways of the Rac and Rho GTPase network (Fig. 5G). Although these findings reflected only changes at the bulk tissue level, they revealed a possible relationship between the ER signaling of Rac and the Rho GTPase network, as well as their female-biased linkages in LOAD subjects. This finding may be useful in explaining how this signaling network is widely suppressed in FDAMic (Additional file 12: Fig. S3A).

### *APOE4* and female sex are risk factors associated with the emergence of FDAMic

**Fig. 6:**
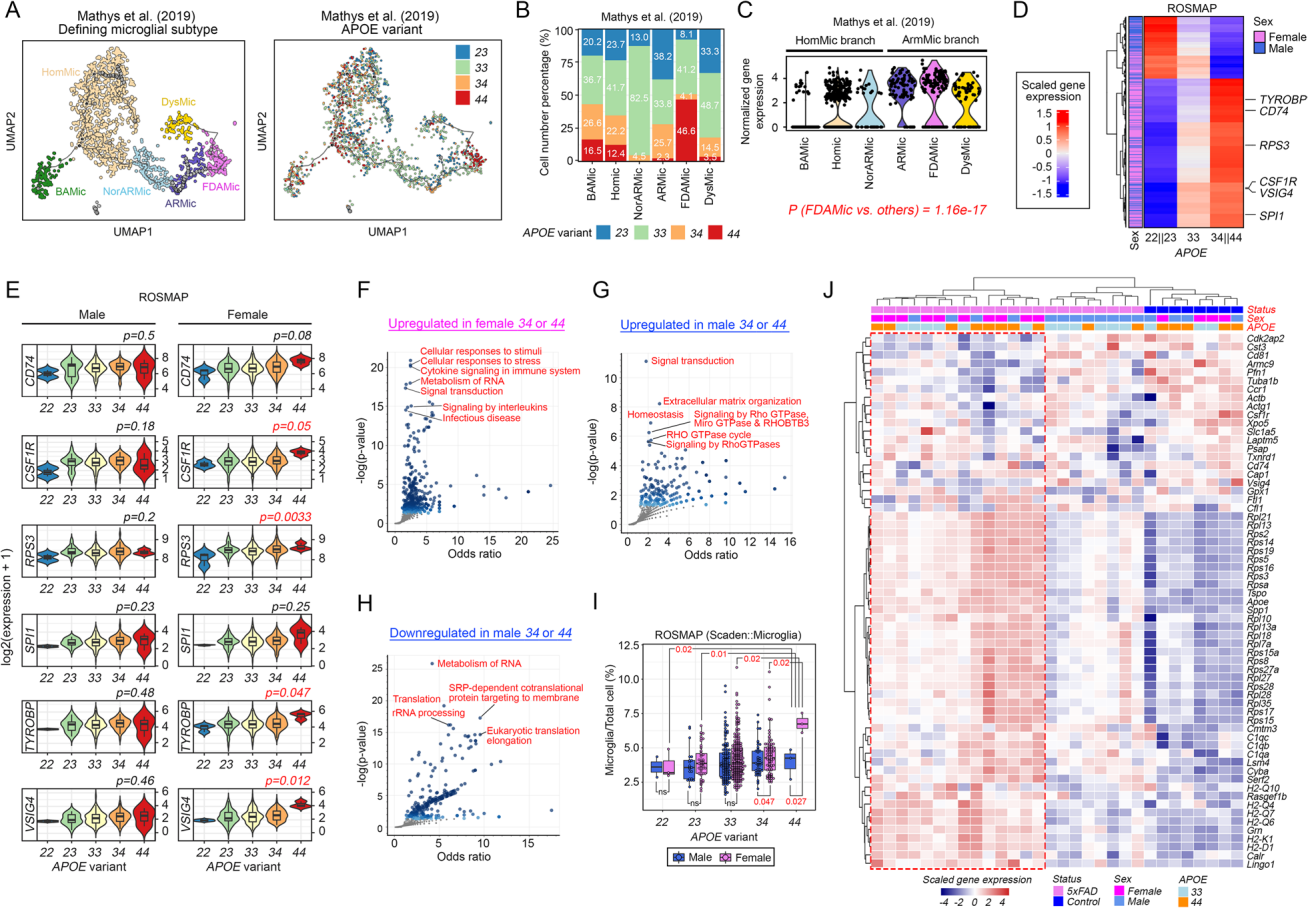
*APOE4* and female sex are risk factors for the emergence of FDAMic. **A** Visualization of *APOE* status distribution in all subtypes of microglia from the Mathys et al. cohort. **B** Ratios of nuclei of different *APOE* statuses in all subclusters of microglia. **C** Violin plot illustrating the normalized expression level of the *APOE* gene in all microglial subtypes. **D** Heatmap illustrating the major trend of changes in gene expression across individuals carrying different combinations of *APOE* allelic variants. **E** Violin plots illustrating the expression profiles of key microglial proliferation-associated genes across samples with different combinations of *APOE* allelic variants. **F–H** Functional enrichment analysis of the **F** upregulated DEGs in female subjects carrying *APOE-34* or APOE-*44* and the **G** upregulated and **H** downregulated DEGs in male subjects carrying *APOE-34* or APOE-*44* compared to subjects of the respective sex with combinations of *APOE-22,* APOE-*23* or APOE-*33*. **I** Cell ratio changes in microglial populations of the ROSMAP samples predicted by the Scaden deep learning algorithm. Comparisons were made across either male or female subjects carrying different combinations of *APOE* allelic variants. **J** Heatmap illustrates the matching of DEGs in FDAMic (Fig. 3A) to transcriptome profiles of microglia harvested from control or 5xFAD mice in which endogenous *ApoE* alleles were replaced by either human *APOE3* (i.e., *33*) or *APOE4* (i.e., *44*)

The menopausal transition and a decline in estrogen hormonal signaling are natural and inevitable changes in all aging women [104]. Therefore, additional risk factors must be in play to confer selective vulnerability to disease pathogenesis among certain individuals. It has been reported previously that a greater penetrance of the *APOE4* genotype in females might exist [105]; therefore, it is speculative that the *APOE* genetic status may also affect the emergence of FDAMic. As presented on a pseudotime trajectory map, FDAMic were uniquely enriched with nuclei from *APOE-44* samples but included the least number of cells from *APOE-23* samples, an allelic combination that was proposed to exhibit disease protective effects (Fig. 6A, B) [106]. Characterization of the *APOE* gene expression level regardless of its variant status revealed that FDAMic expressed the highest level of *APOE* among all subtypes (Fig. 6C), suggesting that FDAMic are likely more affected by defective *APOE4* than other subtypes.

The *APOE* gene encodes a 299 amino acid cell surface glycoprotein that primarily functions as a lipid transporter [107]. However, a previous study indicated that *APOE4* can indirectly affect cellular transcriptomic profiles [108], suggesting that it may facilitate the acquisition of FDAMic transcriptomic signatures in microglia. Using the ROSMAP dataset, gene expression trend analyses across samples belonging to 3 major groups of *APOE* variants (i.e., *22* and *23* versus *33* versus *34* and *44*) in a sex-specific manner were conducted (Fig. 6D). In contrast to observations in male subjects, in whom gene inductions were enriched mainly in subjects of the *APOE22* and *APOE23* background, more extensive inductions in gene expression were found among female subjects of the *APOE34* and *APOE44* background (Fig. 6D, Additional file 9: Table S9). Notably, signature genes of FDAMic, including those implicated in microglial cell proliferation (i.e., *CD74* [99], *CSF1R* [46] and *RPS3* [109]), LOAD pathogenesis (i.e., *TYROBP* [63] and *SPI1* [110]) and immune checkpoint signaling (i.e., *VSIG4* [111]), were the most upregulated in the *APOE34/44* group, specifically among females (Fig. 6E). To understand how these transcriptomic changes manifest into the relevant pathways in different sexes and *APOE* statuses, pathway enrichment analysis was performed. Of the upregulated DEGs curated from the comparison between *APOE34*/*44* females and females without the *APOE4* allele (i.e., *APOE22, 23 and 33*), many were related to stress responses, RNA metabolism, cytokines and interleukin signaling (Fig. 6F). Of the upregulated DEGs curated from similar comparisons made among male subjects, these genes were implicated in extracellular matrix organization, homeostasis and, unexpectedly, multiple pathways of the Rho GTPase signaling network (Fig. 6G). The absence of an activated Rho GTPase network in female *APOE34*/*44* brain samples suggested that their microglia are likely more vulnerable to extra insults that further downregulate this signaling network and its associated phagocytic activities, which resembles FDAMic. Additionally, a set of downregulated DEGs was found in male *APOE34*/*44* samples, which was associated with suppressed RNA metabolism and a protein translation network (Fig. 6H) that is deemed to be neuroprotective, as these changes may suppress immunosenescence [112, 113]. Quantitatively, female subjects carrying even a single *APOE*4 allele were associated with significant increments in total microglial populations compared to non*-APOE4* carriers (i.e., 22, 23 and 33) of the same sex or male subjects of the same *APOE34*/*44* background (Fig. 6I), which could be at least in part caused by the emergence of FDAMic.

To further validate the importance of *APOE4* as a partnering risk factor for female sex that promotes the emergence of FDAMic during AD pathogenesis, a matching analysis with the list of upregulated DEGs found in FDAMic (Fig. 3A) was performed against a mouse microglia dataset, from which microglia were harvested from either control or 5xFAD mice of both sexes that had their endogenous mouse *ApoE* alleles replaced by either the humanized *APOE3* (i.e., *33*) or *APOE4* (i.e., *44*) orthologs [114]. Consistent with the predictions in human brain samples, the gene expression patterns that most closely matched those of FDAMic were found in microglia harvested from female mice (i.e., 10/14 = 71.4% mice on the left branch of the hierarchical clustering) or from those with the *APOE-44* genotype (i.e., 8/14 = 57.1% mice on the left branch) (Fig. 6J). Notably, among the latter, 5 (out of 7 in total in the entire study cohort, i.e., 5/7 = 71.4%) were female *APOE44* mice (Fig. 6J). Intriguingly, these 5 mice were also 5XFAD (i.e., the remaining 2 out of 7 female *APOE44* mice were non-5XFAD on the right branch of the hierarchical cluster) (Fig. 6J), suggesting that Aβ pathologies may stand alone as a risk factor contributing to FDAMic signatures. However, the majority of microglia from male 5XFAD mice (i.e., 8/12 = 66.6%), regardless of their *APOE* status, failed to exhibit strong FDAMic signatures (Fig. 6J, right branch of the hierarchical cluster). Together, these discrepancies highlighted that female sex in combination with *APOE44* may formulate a strong set of risk factors favoring the emergence of FDAMic.

## Discussion

Sex dimorphism in microglial function and neuroinflammation is implicated in AD pathogenesis. Our study revealed that microglia are the only detected cell type that exhibited sex differences in their relative cell quantities during the process of LOAD pathogenesis, characterized by more pronounced inductions in number in affected female subjects as the disease progresses. Compared to the respective sex-matched nondementia controls, more profound changes in transcriptomic profiles were also observed in female subjects with LOAD. These observations agree with previous conclusions drawn from mouse studies [17, 115], supporting that microglia are the key cell type that contributes to sex dimorphic changes observed in the disease.

Microglia constitute a heterogeneous cell population, and changes in composition among various subtypes of microglia may greatly affect brain neuroimmune homeostasis and vulnerability to different neurodegenerative diseases. Our analyses of different subtypes of microglia confirmed the unique existence of FDAMic enriched in affected female subjects, particularly among *APOE4* carriers. Quantitatively, the relative cell number ratio of FDAMic is positively associated with more advanced disease pathologies. The relevance of FDAMic to the sex-biased differences observed in the global microglial population was also confirmed, as selective exclusion of their nuclei from the analyses greatly abolished the observations.

Compared to the rest of the microglial populations, FDAMic likely exhibit stronger cell proliferative properties, as defined by Friedman et al. [70]. Moreover, their transcriptomic signature also revealed higher expression levels of MHC class II autoantigens and Aβ binding receptor genes. It was previously reported that MHC class II autoantigen-expressing microglia were associated with various autoimmune-related neurodegenerative diseases and the development of chronic inflammatory lesions [116–119], which hinted at an intrinsic pathological nature of these cells. In addition to these gain-of-function genes, downregulated genes in FDAMic also suggested a loss in phagocytic activities, which may render them ineffective in clearing Aβ and other protein aggregates from the region [120] despite exhibiting Aβ binding properties. Together, these phenotypic characteristics suggest that these cells are quantitatively associated with more severe pathologies of the disease.

Mechanistically, our analyses suggested that loss of ER signaling is associated with, and possibly in part underlies, the emergence of FDAMic. Among other subtypes of microglia, FDAMic exhibit the lowest expression level of *ESR1* or *ESR2,* and therefore, they are more likely to be defective in ER signaling, which is known to confer anti-inflammatory properties to microglia [121]. Our analyses revealed that ER signaling supports the activities of multiple transcription regulons that take part in shaping the homeostatic properties of HomMic, ARMic and BAMic by interacting with key transcription factors activated in these cells. Some of these transcription factors are gene targets of *ESR1*/ERα as well. On the other hand, ER signaling may modulate the global status of DNA methylation at gene regulatory regions such that the accessibility of transcription factors to these sites is altered. Our analyses revealed that many of these ER signaling-regulated DNA methylation gene targets are implicated in the Rac and Rho GTPase signaling network, which is known to be crucial in supporting microglial phagocytosis [122]. In LOAD, our analysis indicated that the majority of the genes were hypermethylated in affected females. Although the bulk tissue DNA methylome data failed to directly provide insights at single-cell resolution to pinpoint the changes to microglia, the identification of this linkage between ER signaling and DNA methylation targets suggested that the diminishing sex hormone signals that occur during the menopausal period may reshape the brain microenvironment that favors the emergence of FDAMic.

The menopausal transition and a decline in ER signaling are inevitable in all women [104]. Therefore, we reasoned that additional risk factors must underlie selective disease vulnerability in affected individuals. Here, we report that the coexistence of *APOE4* genetic status—the strongest genetic risk factor for LOAD [123]—with female sex formulates a set of strong risk factors favoring the emergence of FDAMic. Since FDAMic are associated with more advanced pathologies and cognitive decline, their emergence explains, at least in part, why a greater penetrance effect is found among female carriers of *APOE4* [124]. In the clinic, hormone replacement therapy (HRT) is used as a strategy to mitigate cognitive decline during menopausal transition and postmenopausal periods. The sex- and *APOE4*-biased trigger for the emergence of FDAMic may explain how HRT therapy is associated with higher efficacies among *APOE4* carriers [124]. It has been suggested that HRT should be administered early in the initiation of the menopausal transition to achieve a better protective effect. It is plausible that this beneficial effect is in part mediated by sustaining the ER signaling network in ARMic, which therefore prevents their subsequent transformation into FDAMic along the cell fate trajectory—a pathogenic and dysfunctional subtype of microglia. Nevertheless, this nature of FDAMic suggested that selective modulation of these cells and their precursors in the brain may help to regain neuroimmune homeostasis and therefore are a potential new target for future drug development.

While this work presents a set of in-depth analyses conducted with datasets from multiple sources to address how female sex may affect the relative quantities of microglial subpopulations and whether changes in the fidelity of estrogen receptor (ER) signaling may remodel the properties of these cells, there are still limitations to our study. One major limitation is the relatively low ratio of microglia in the brain, which renders further exploration on how these cells vary across different stages of LOAD challenging. This issue could be resolved by utilizing datasets of large sample sizes that were recently made available to the research community. Examples include the datasets associated with the Sun et al. [20] and Green et al. [21] (a preprint manuscript) studies. Our preliminary analyses with their supplementary data indicated that the lipid-associated/processing microglial subtype highlighted in these studies is indeed enriched in female and disease-associated nuclei (Additional file 13: Fig. S4, Additional file 14: Fig. S5), and their corresponding marker genes (i.e., *PPARG*^+^*, APOE*^+^*, TREM*2^+^) are also highly expressed in FDAMic (Additional file 13: Fig. S4A). This indirectly validated the existence of a population of “FDAMic-like” microglia in these independent datasets, and whether they truly resemble the molecular signature of FDAMic warrants future investigation. Furthermore, according to the paper by Keren-Shaul et al. [16], activation of disease-associated microglia (DAM) is characterized by induced phagocytic and lipid metabolic activities via upregulated expression of *APOE, LPL, CD9, CDY7* and *TREM2* and concurrent downregulation of microglia checkpoint genes (e.g., *CX3CR1*) [16]. Considering that FDAMic also exhibited the highest expression levels of *APOE* and *TREM2* (Additional file 13: Fig. S4A) but the lowest expression level of *CXCR3*1 (Fig. 2C) among other subtypes of microglia, it is therefore speculated that FDAMic resembles the activated state of DAM, while their predominant *APOE4* and female sex status (Fig. 6) might have hindered the activation of phagocytic activities through the Rac and Rho GTPase signaling network [125–128] and associated autophagy pathways [129] (Additional file 12: Fig. S3A). However, the original DAM properties were mainly based on previous mouse studies [16] and are substantially different from microglial signatures identified in human AD brains [130]. This discrepancy could be due to the fact that these cells only constituted a small number of nuclei in the entire microglial population of the original mouse study [16], and greater variabilities exist in human versus mouse samples [16]. Consistent with this, a recent study also indicated that DAM-like signatures in the human LOAD brain do not encompass one single state but rather multiple substates [20]. Further speculation shall therefore proceed with caution. Nevertheless, if our prediction of FDAMic as a subgroup of lipid-processing DAMs is correct, this would offer important insights into how the *APOE* genetic status and sex of the subject may alter the lipid-processing properties and neuroprotective nature of activated DAMs and will be validated in future studies.

### Supplementary Information

**Additional file 1: Table S1.** Sample information from Mathys et al. (2019) (single-nucleus RNA-seq), Lau et al. (single-nucleus RNA-seq), Marabito et al. (2021) (single-nucleus RNA-seq), Green et al. (2023) (single-nucleus RNA-seq), Sun et al. (2023) (single-nucleus RNA-seq) and ROSMAP (both transcriptomic and DNA methylomic data) studies.

**Additional file 2: Table S2.** DEGs from the ROSMAP study identified using the limma method.

**Additional file 3: Table S3.** DEG lists and numbers found between LOAD and ND (1) before and after (2) FDAMic or (2) random nuclei removal.

**Additional file 4: Table S4.** A total of 373 DEGs were identified in FDAMic versus the rest of the microglia in the Mathys et al. dataset.

**Additional file 5: Table S5.** Protein‒protein interacting partners of ESR1/ERα and ESR2/ERβ extracted from the STRING and PPI network of the BIOGRID database.

**Additional file 6: Table S6.** 288 SCENIC-identified *SPI1*/PU.1 target genes.

**Additional file 7: Table S7.** 1,125 estrogen-responsive genes identified from the DNA methylome profiles in the MCF-7 study.

**Additional file 8: Table S8.** Hypermethylated and hypomethylated genes found in brain tissues of female LOAD patients.

**Additional file 9: Table S9.** Data table for heatmap visualization of gene expression trends in various APOE allelic variants.

**Additional file 10: Figure S1.** Additional data supporting microglial populations are uniquely altered in affected females. A. T-SNE plots label all cell types in the Mathys et al. cohort by various validated markers. *NGRN* for excitatory neuron; *GAD1* for inhibitory neuron; *MBP* for oligodendrocyte; *GFAP* for astrocyte; *FTL1* for endothelial cell; *CSF1R* for microglia and *VCAN* for oligodendrocyte precursor cell. B. T-SNE plot clustering of all cell types from the Lau et al. cohort. Different subclusters are labeled by different color and number codes. Validated marker genes, including *CD74, CSF1R* and *P2RY12,* were used to identify microglia. C. Relative Cluster 6 cell ratio changes in ND versus LOAD samples of different sexes from the Lau et al. cohort. D. T-SNE plot labeling all cell types from the Mathys et al. cohort indicates that the male versus female nuclei distributions in ND, early and late disease status were similar. E–F. Cell ratio changes of Cluster 13 from the Mathys et al. cohort relative to E Braak staging and F Cerad scores. G. Relative cell ratio changes in microglial populations in the ROSMAP samples predicted by the CIBERSORTx deconvolution method (top) or Scaden deep learning (middle and bottom) algorithm. Comparisons were made according to disease diagnosis (top), Braak staging (middle) or Cerad score (bottom). H. Violin plots illustrate changes in the expression levels of microglia-enriched genes (i.e., *CSF1R* and *CD81*) in ND and LOAD samples of different sexes. I. Normalized expression levels of the *CSF1R* gene in Cluster 13 of the Mathys et al. cohort. Comparisons were made according to sex and Cogdx scores of the samples. J. Mapping of sex-specific DEGs (left table: LOAD versus NS; right table: Early disease/Mild cognitive impairment versus ND) in Cluster 13 of the Mathys et al. cohort to KEGG pathways.

**Additional file 11: Figure S2.** Additional data supporting the identification and characterization of FDAMic. A. Unsupervised clustering of all microglial subclusters identified. B. Cell differentiation status estimation among different subclusters of microglia by the SCENT entropy-based method (top) or the CytoTRACE algorithm (bottom). C. Heatmap illustrating the normalized expression levels of marker genes of different microglial subclusters. D-E. Visualization of all subtypes of microglia on the evolutionary trajectory UMAP plot according to D cognitive impairment scores or E age of the samples. F. Scaled brain sample number distribution from the Mathys et al. dataset in different subclusters of microglia. G. Top: UMAP plot labeling all cell types from the Morabito et al. cohort. Bottom: UMAP plots indicate the cluster location of microglia by common marker genes, including *CX3CR1, CD74, P2RY12* and *CSF1R*. H. Violin plots of key microglial marker genes illustrating the interrelationship between FDAMic and other microglial subtypes. I. Volcano plot illustrating DEGs identified in FDAMic compared to the rest of the microglial population in the Mathys et al. cohort. J. Box plot displaying the effect on DEG significance presented in Fig. 1M when FDAMic nuclei or equal quantities of random nuclei were eliminated. K. Violin plots illustrate changes in the expression levels of microglia-enriched genes (i.e., *RPS19*, *CSF1R* and *APOE*) in ND and LOAD samples of different sexes from the Mathys et al. cohort after selective elimination of FDAMic from the analysis.

**Additional file 12: Figure S3.** Additional data for illustrating the molecular changes in FDAMic. A. Over‐representation analysis (ORA) of downregulated DEGs in FDAMic presented in Fig. 3A using the Metascape platform. Every node represents an enriched term, and two nodes are linked if their Kappa similarities were higher than 0.3. Similar functional terms are clustered together and are displayed using the same color. Node size is proportional to the number of enriched genes. B-C. Dot plots present the average expression levels of uniquely (B) up- and (C) downregulated DEGs found in FDAMic. D-M. GO pathway enrichment analysis of genes enriched in significantly altered KEGG pathways in FDAMic (i.e., those highlighted in red and blue in Fig. 3D).

**Additional file 13: Figure S4.** Subclusters of lipid-associated microglia (i.e., Mic.12–13) defined by the Green et al. [21] study are enriched with female and disease-associated nuclei. A. Expression levels of the *APOE*, *TREM2* and *PPARG* genes in various microglial subtypes defined by our study based on the Mathys et al. discovery dataset [22]. B. The disease status of samples from the Green et al. study [21] was defined based on multiple clinicopathological parameters (x-axis). C. Microglial cell fraction distribution in LOAD pathological groups in different sexes. Subclustering was defined by Green et al. (2023) [21].

**Additional file 14: Figure S4.** A subcluster of microglia with enhanced lipid-processing properties (i.e., MG4) defined by the Sun et al. study [20] is enriched with female and disease-associated nuclei. Microglial cell fraction distribution in LOAD versus nondemented groups of different sexes. Subclustering was defined by Sun et al. (2023) [20].

## Data Availability

The datasets used and/or analyzed during the current study are available from the corresponding author upon reasonable request. All analyses were carried out using freely available software packages. All original codes for each figure can be found at https://github.com/KimChow-Lab/FDAMic.

## Abbreviations

AgedMic: Aged microglia phenotype
APOE: Apolipoprotein
ARMic: Activated response microglia
Aβ: Amyloid beta
BAMic: Border-associated macrophage-like microglia
DAM: Disease-associated microglia
DysMic: Disease-associated dystrophic microglia
ER: Estrogen receptor
FDAMic: Female-enriched and disease-associated microglia
GSEA: Gene set enrichment analysis
GO: Gene Ontology
LOAD: Late-onset Alzheimer’s disease
MGnD: Microglial neurodegenerative phenotype
MHC: Major histocompatibility complex
NeuroDegen: Neurodegenerative disease phenotype
NES: Normalized enriched score
NFT: Neurofibrillary tangle
NorARMic: ARMic enriched with nuclei from normal, nondementia samples.
RPS19: Ribosomal protein S19
ROSMAP: Religious Orders Study and Memory and Aging Project
