# Supplementary figures and images for: Identification of female-enriched and disease-associated microglia (FDAMic) contributes to sexual dimorphism in late-onset Alzheimer’s disease
Source: J Neuroinflammation. 2024 Jan 4;21:1. doi: 10.1186/s12974-023-02987-4 (PMC10765928; doi:10.1186/s12974-023-02987-4)

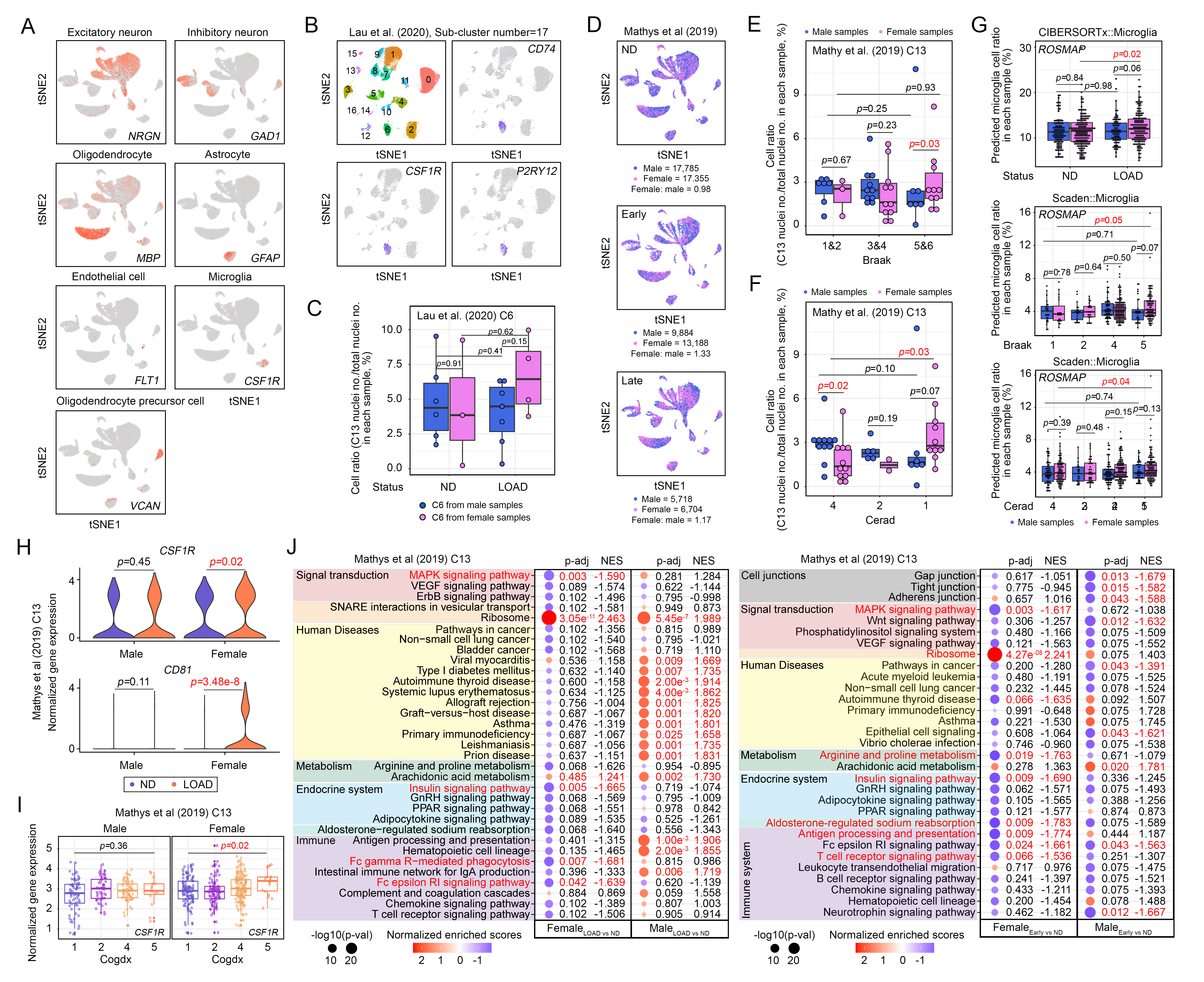

Supplement: Supplementary file 10 — Additional file 10: Figure S1. Additional data supporting microglial populations are uniquely altered in affected females. A. T-SNE plots label all cell types in the Mathys et al. cohort by various validated markers. NGRN for excitatory neuron; GAD1 for inhibitory neuron; MBP for oligodendrocyte; GFAP for astrocyte; FTL1 for endothelial cell; CSF1R for microglia and VCAN for oligodendrocyte precursor cell. B. T-SNE plot clustering of all cell types from the Lau et al. cohort. Different subclusters are labeled by different color and number codes. Validated marker genes, including CD74, CSF1R and P2RY12, were used to identify microglia. C. Relative Cluster 6 cell ratio changes in ND versus LOAD samples of different sexes from the Lau et al. cohort. D. T-SNE plot labeling all cell types from the Mathys et al. cohort indicates that the male versus female nuclei distributions in ND, early and late disease status were similar. E–F. Cell ratio changes of Cluster 13 from the Mathys et al. cohort relative to E Braak staging and F Cerad scores. G. Relative cell ratio changes in microglial populations in the ROSMAP samples predicted by the CIBERSORTx deconvolution method (top) or Scaden deep learning (middle and bottom) algorithm. Comparisons were made according to disease diagnosis (top), Braak staging (middle) or Cerad score (bottom). H. Violin plots illustrate changes in the expression levels of microglia-enriched genes (i.e., CSF1R and CD81) in ND and LOAD samples of different sexes. I. Normalized expression levels of the CSF1R gene in Cluster 13 of the Mathys et al. cohort. Comparisons were made according to sex and Cogdx scores of the samples. J. Mapping of sex-specific DEGs (left table: LOAD versus NS; right table: Early disease/Mild cognitive impairment versus ND) in Cluster 13 of the Mathys et al. cohort to KEGG pathways. [file 12974_2023_2987_MOESM10_ESM.tif]

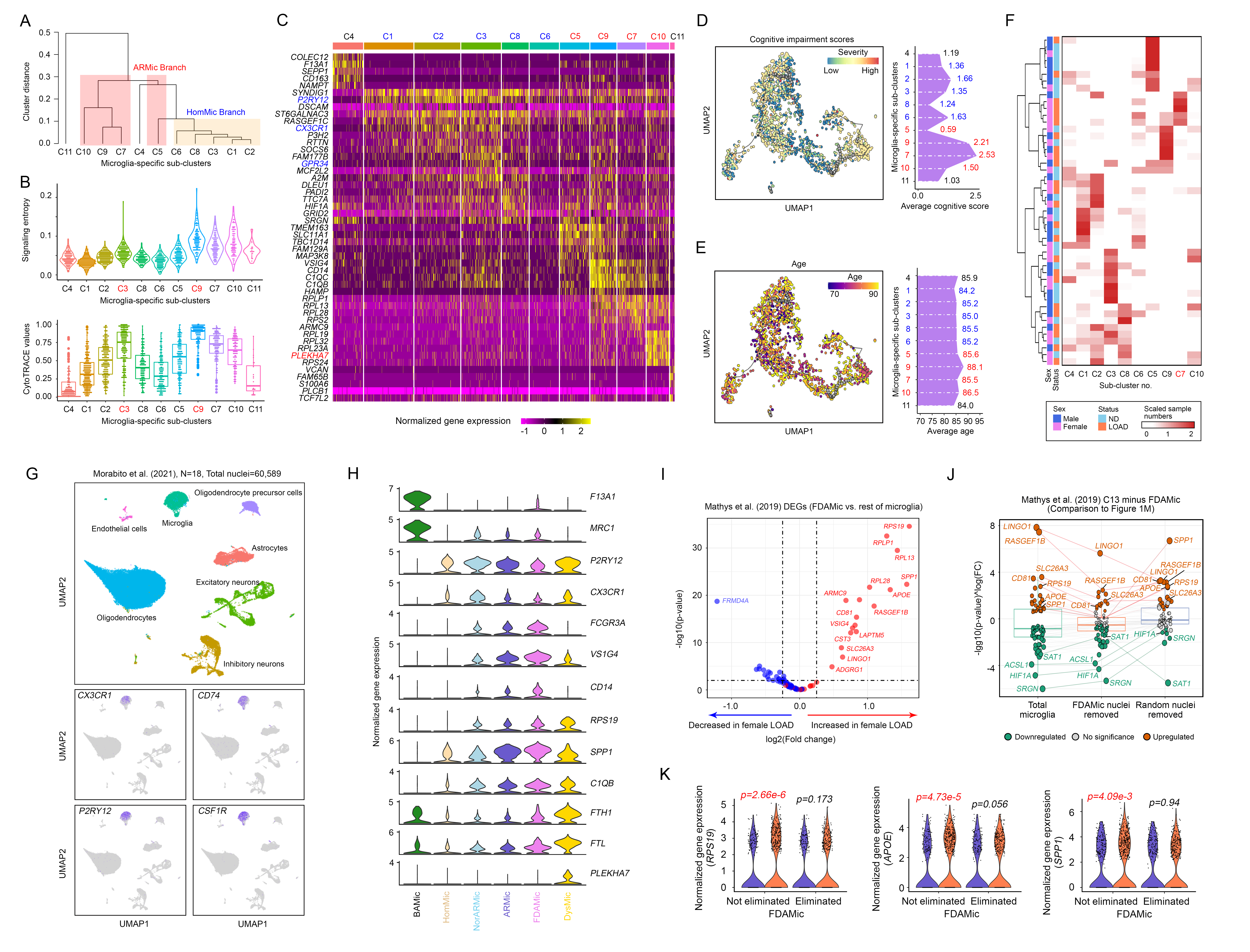

Supplement: Supplementary file 11 — Additional file 11: Figure S2. Additional data supporting the identification and characterization of FDAMic. A. Unsupervised clustering of all microglial subclusters identified. B. Cell differentiation status estimation among different subclusters of microglia by the SCENT entropy-based method (top) or the CytoTRACE algorithm (bottom). C. Heatmap illustrating the normalized expression levels of marker genes of different microglial subclusters. D-E. Visualization of all subtypes of microglia on the evolutionary trajectory UMAP plot according to D cognitive impairment scores or E age of the samples. F. Scaled brain sample number distribution from the Mathys et al. dataset in different subclusters of microglia. G. Top: UMAP plot labeling all cell types from the Morabito et al. cohort. Bottom: UMAP plots indicate the cluster location of microglia by common marker genes, including CX3CR1, CD74, P2RY12 and CSF1R. H. Violin plots of key microglial marker genes illustrating the interrelationship between FDAMic and other microglial subtypes. I. Volcano plot illustrating DEGs identified in FDAMic compared to the rest of the microglial population in the Mathys et al. cohort. J. Box plot displaying the effect on DEG significance presented in Fig. 1M when FDAMic nuclei or equal quantities of random nuclei were eliminated. K. Violin plots illustrate changes in the expression levels of microglia-enriched genes (i.e., RPS19, CSF1R and APOE) in ND and LOAD samples of different sexes from the Mathys et al. cohort after selective elimination of FDAMic from the analysis. [file 12974_2023_2987_MOESM11_ESM.tif]

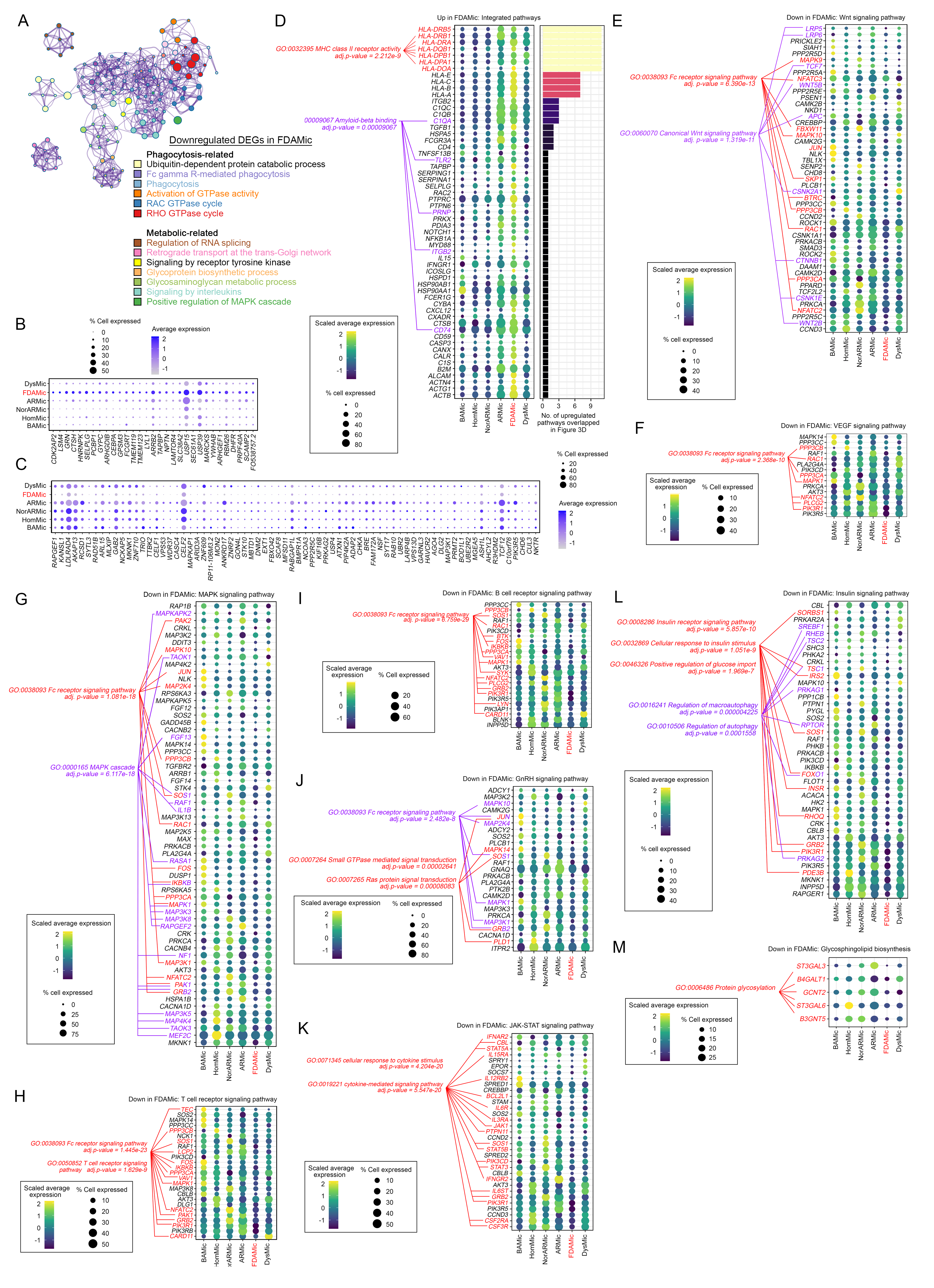

Supplement: Supplementary file 12 — Additional file 12: Figure S3. Additional data for illustrating the molecular changes in FDAMic. A. Over‐representation analysis (ORA) of downregulated DEGs in FDAMic presented in Fig. 3A using the Metascape platform. Every node represents an enriched term, and two nodes are linked if their Kappa similarities were higher than 0.3. Similar functional terms are clustered together and are displayed using the same color. Node size is proportional to the number of enriched genes. B-C. Dot plots present the average expression levels of uniquely (B) up- and (C) downregulated DEGs found in FDAMic. D-M. GO pathway enrichment analysis of genes enriched in significantly altered KEGG pathways in FDAMic (i.e., those highlighted in red and blue in Fig. 3D). [file 12974_2023_2987_MOESM12_ESM.tif]

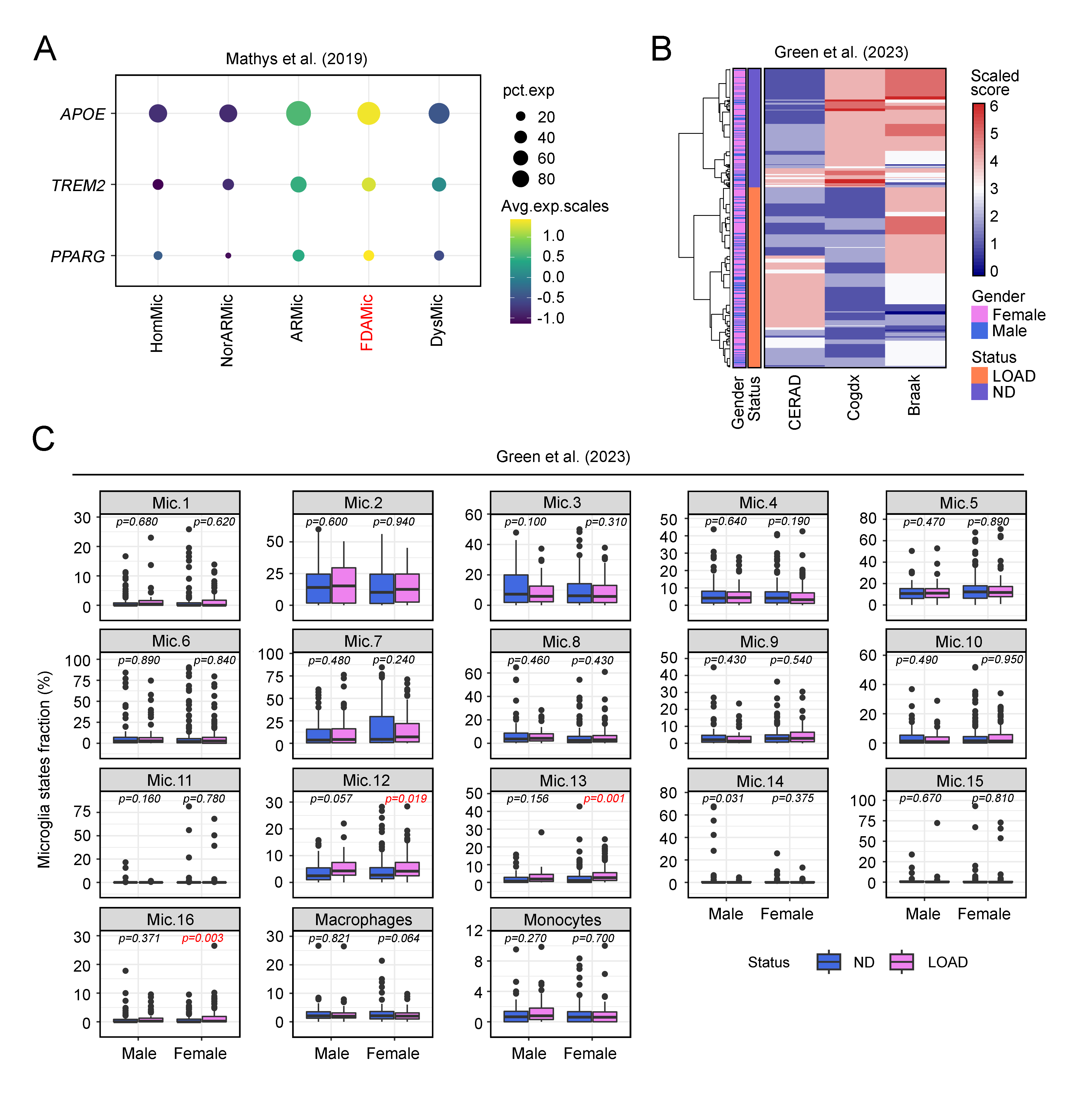

Supplement: Supplementary file 13 — Additional file 13: Figure S4. Subclusters of lipid-associated microglia (i.e., Mic.12–13) defined by the Green et al. [21] study are enriched with female and disease-associated nuclei. A. Expression levels of the APOE, TREM2 and PPARG genes in various microglial subtypes defined by our study based on the Mathys et al. discovery dataset [22]. B. The disease status of samples from the Green et al. study [21] was defined based on multiple clinicopathological parameters (x-axis). C. Microglial cell fraction distribution in LOAD pathological groups in different sexes. Subclustering was defined by Green et al. (2023) [21]. [file 12974_2023_2987_MOESM13_ESM.tif]

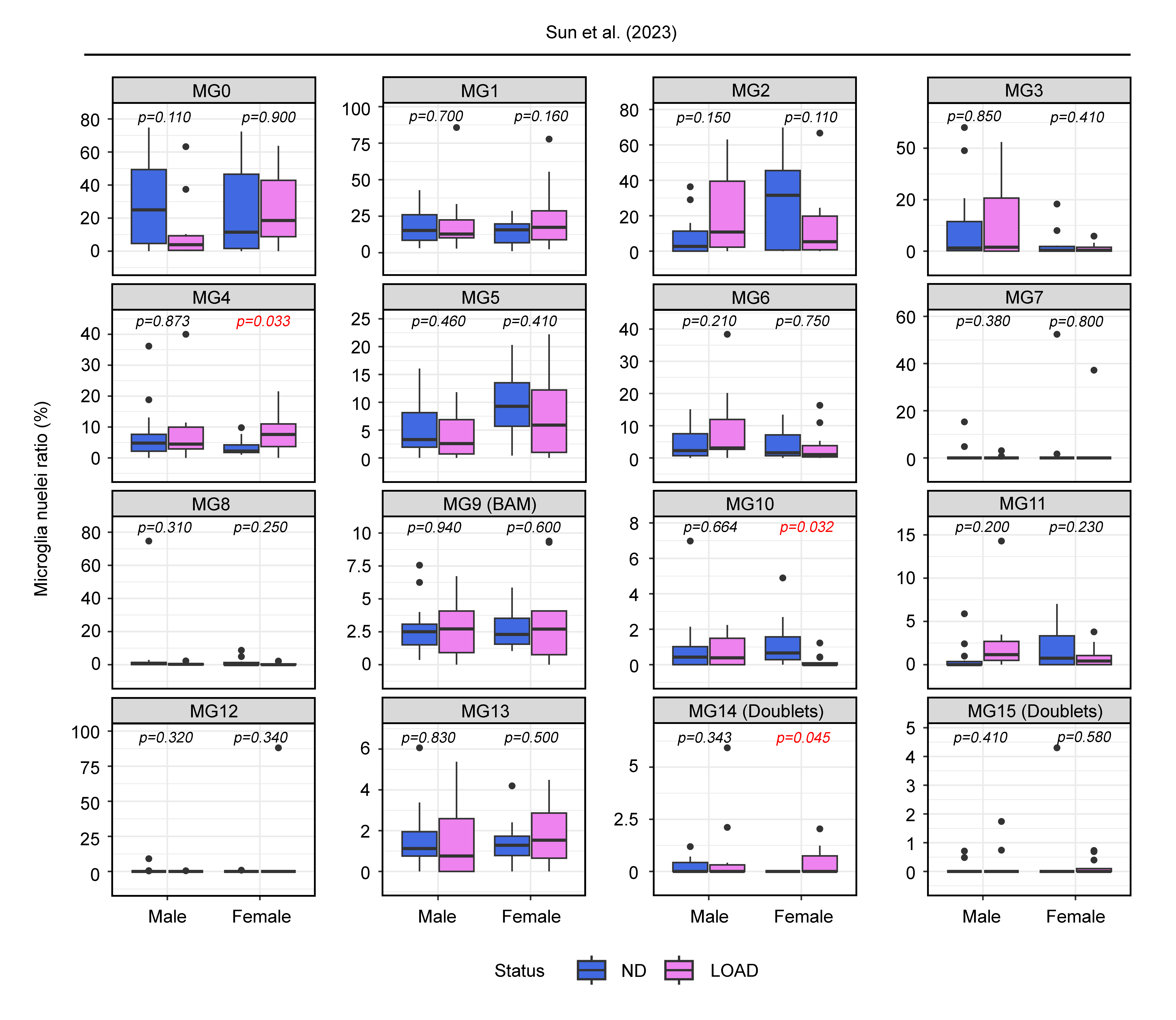

Supplement: Supplementary file 14 — Additional file 14: Figure S4. A subcluster of microglia with enhanced lipid-processing properties (i.e., MG4) defined by the Sun et al. study [20] is enriched with female and disease-associated nuclei. Microglial cell fraction distribution in LOAD versus nondemented groups of different sexes. Subclustering was defined by Sun et al. (2023) [20]. [file 12974_2023_2987_MOESM14_ESM.tif]
